# Establishment and Characterization of a Highly Tumourigenic and Cancer Stem Cell Enriched Pancreatic Cancer Cell Line as a Well Defined Model System

**DOI:** 10.1371/journal.pone.0048503

**Published:** 2012-11-12

**Authors:** Johannes Fredebohm, Michael Boettcher, Christian Eisen, Matthias M. Gaida, Anette Heller, Shereen Keleg, Jörg Tost, Karin M. Greulich-Bode, Agnes Hotz-Wagenblatt, Mark Lathrop, Nathalia A. Giese, Jörg D. Hoheisel

**Affiliations:** 1 Functional Genome Analysis, German Cancer Research Center (DKFZ), Heidelberg, Germany; 2 Stem Cells and Cancer, German Cancer Research Center (DKFZ), Heidelberg, Germany; 3 Institute of Pathology, University Hospital of Heidelberg, Heidelberg, Germany; 4 European Pancreas Center, University Hospital of Heidelberg, Heidelberg, Germany; 5 Institut de Génomique, Centre National de Génotypage, Evry, France; 6 Genetics of Skin Carcinogenesis, German Cancer Research Center (DKFZ), Heidelberg, Germany; 7 Functional Proteome Analysis, German Cancer Research Center (DKFZ), Heidelberg, Germany; University of Illinois at Chicago, United States of America

## Abstract

Standard cancer cell lines do not model the intratumoural heterogeneity situation sufficiently. Clonal selection leads to a homogeneous population of cells by genetic drift. Heterogeneity of tumour cells, however, is particularly critical for therapeutically relevant studies, since it is a prerequisite for acquiring drug resistance and reoccurrence of tumours. Here, we report the isolation of a highly tumourigenic primary pancreatic cancer cell line, called JoPaca-1 and its detailed characterization at multiple levels. Implantation of as few as 100 JoPaca-1 cells into immunodeficient mice gave rise to tumours that were histologically very similar to the primary tumour. The high heterogeneity of JoPaca-1 was reflected by diverse cell morphology and a substantial number of chromosomal aberrations. Comparative whole-genome sequencing of JoPaca-1 and BxPC-3 revealed mutations in genes frequently altered in pancreatic cancer. Exceptionally high expression of cancer stem cell markers and a high clonogenic potential *in vitro* and *in vivo* was observed. All of these attributes make this cell line an extremely valuable model to study the biology of and pharmaceutical effects on pancreatic cancer.

## Introduction

Pancreatic cancer is one of the most aggressive types of cancer. Mortality is nearly identical to incidence. With a five-year survival rate of less than 5%, it is the fourth most common cause of cancer-related deaths in the developed world [1]. Reasons for the poor prognosis are the late clinical diagnosis [2] and the tumour's resistance to standard chemotherapy [3]. With about 90% of the cases, pancreatic ductal adenocarcinoma (PDAC) is the most common form and responsible for the high mortality; other, rare cancer subtypes, such as cystic tumours, are less lethal. PDAC is assumed to arise from epithelial cells of the pancreatic ductal system [4]. Distribution of tumour cells within the malignant tissue is typically diffuse and contains areas with varying degrees of histological differentiation. It is also characterized by an overall heterogeneous tumour cell population (intratumoural heterogeneity) [5], [6].

A substantial number of PDAC cell lines of different characteristics have been established and provide a cellular source for investigating molecular aspects of this devastating disease (comprehensively reviewed by Ulrich et al. [7]). However, culturing cell lines under well-defined conditions inevitably leads to the selection of subpopulations over time. Thus, standard cell lines do not model the situation of intratumoural heterogeneity, since clonal selection has taken place over many passages *in vitro*. This leads to a homogeneous population of cells by genetic drift [8]. On the contrary, primary cell lines very closely resemble the heterogeneity of the primary tumour and therefore provide a good resource for cell culture experiments.

The *in vivo* heterogeneity of tumour cells is especially critical for experiments testing the effects of chemotherapy, because heterogeneity provides the foundation for the selection of resistant subpopulations, which in turn forms the basis for acquired drug resistance and reoccurrence of the tumour [3], [9]. Among the population of resistant cells, cancer stem cells play a particularly crucial role as they possess the ability to self-renew [3] and are able to repopulate the tumour at the primary site or lead to metastasis formation at distant sites [10].

Cancer stem cells or tumour initiating cells that are driving tumour progression have received a lot of attention in the last decades [11], [12], [13]. For pancreatic cancer, several molecular markers have been suggested to be characteristic for cancer stem cells. Among these are expression of the triplet CD44, CD24 and ESA [14], the cell surface protein CD133 (prominin I) [15], [16], [17], and - more recently - increased activity of aldehyde dehydrogenase 1 (ALDH1) [18]. Expression of ALDH1 has also been correlated with invasion and migration as well as mesenchymal features of the tumour [19]. Moreover, it has been associated with poor overall survival. Interestingly, however, two conflicting studies show that high [19] or low [20] expression of ALDH1, has an adverse effect on patient survival. Another hallmark of tumour initiation is the ability to form spheroids or tumour spheres in suspension [21], [22]. Cancer stem cells are also believed to contribute to the development of drug resistance. Concurrently, it has been shown that the content of CD133 expressing cells can be enriched by gemcitabine treatment [23], [24], [25] suggesting a pivotal role of this CSC marker in gemcitabine resistance.

Here, we report the isolation and characterisation of a new primary cell line derived directly from a human tumour sample of a patient with pancreatic ductal adenocarcinoma. This cell line, named JoPaca-1, exhibits a high variety in morphology and carries many chromosomal aberrations. Pathologically, mouse-xenografts and the primary tumour have a high degree of similarity in terms of diffusive differentiation and infiltration of surrounding pancreatic and non-pancreatic tissue. JoPaca-1 cells express the tumour marker mesothelin and several cytokeratins. Apart from its high heterogeneity, probably the most important feature of this new cell line is its high content of tumour initiating cells as demonstrated by *in vivo* limiting dilution assay and high expression levels of cancer stem cell markers. In view of its close resemblance to the original tumour and in combination with the thorough molecular characterisation, the cell line provides an excellent resource for any kind of cell-based rather than tissue-based analysis.

## Materials and Methods

### Ethics statement

Informed written consent was obtained from the patient. Ethical approval was obtained from the ethics commission of the medical faculty of the University Hospital of Heidelberg, Germany. All animal care and procedures followed German legal regulations and were previously approved by the governmental review board of the state of Baden-Wuerttemberg, Germany.

### Cell isolation

Fresh tissue was obtained from a 46-year old male patient suffering from pancreatic cancer, who underwent a pylorus-preserving pancreatecto-duodenectomy. During the operation, a sample was taken from the removed tumour and directly stored in Hank's balanced salts solution (HBSS, H6648, Sigma Aldrich, Taufkirchen, Germany) at 4°C for 12 hours before further processing. The tissue sample was then cut in pieces of about 5 mm diameter and transferred to a culture flask containing Ham's/F-12 Medium (21765-029, Life Technologies, Darmstadt, Germany), supplemented with serum replacement reagent (S0638, Sigma Aldrich). The tissue pieces attached to the substrate and cells grew out after one week, forming clusters around the tissue fragments. Fibroblasts and stellate cells were removed by two rounds of serial enzymatic detachment with 0.05% Trypsin/EDTA (25300054, Life Technologies). The resulting population of tumour cells was frozen at passage number four and stored in aliquots. For the experiments described here, JoPaca-1 cells were used in passages 6 to 19. Immortalization of the isolated cells was not necessary.

### Cell culture

The well-established pancreatic cancer cell lines MiaPaCa-2, MDAPanc-28, BxPC-3, AsPC-1, and Capan-1 were obtained from ATCC (Rockville, MD, USA). Additionally, FamPAC were kindly provided by Professor Eisold [26] and HPDE c7 cells by Professor Francisco Real (Spanish National Cancer Research Centre, CNIO) [27]. BxPC-3 was authenticated by the German Collection of Microorganisms and Cell Cultures (DSMZ, Braunschweig, Germany). All cell lines were free of mycoplasms, viruses and cell line contamination as tested by PCR [28].

Cells were cultured in standard medium and supplements, which were obtained from Life Technologies, Darmstadt, Germany unless specified otherwise: JoPaca-1 in Isocove's Modified Dulbecco's Medium (IMDM) (Cat. No. 21056), 10% fetal calve serum (FCS), and 1% penicillin-streptomycin (P/S); BxPC-3 in Roswell Park Memorial Institute (RPMI) 1640 medium (Cat. No. 21875), 10% FCS, and 1% PS; HPDE c7 in Keratinocyte Serum-Free Medium (KSFM), 0.15 ng/ml epidermal growth factor receptor, and 25 µg/ml bovine pituitary extract (Cat. No. 17005075) [29]. For tumour sphere formation, JoPaca-1 cells were cultured in Ham's/F12-medium supplemented with 20 ng/ml basic fibroblast growth factor (bFGF, F0291, Sigma Aldrich, Munich, Germany), 1× B-27 supplements (Cat. No. 0080085-SA), 2 mM L-glutamine (Cat. No. 25030024), and 1% P/S in low attachment plates (Cat. No. 3473, Corning, Amsterdam, The Netherlands). Cells were split at a ratio of 1 to 10 unless stated otherwise. Cell viability after treatment with gemcitabine (G6423, Sigma Aldrich) was determined by resazurine assay [30]. For long-term treatment with gemcitabine, JoPaca-1 cells in passage 10 were subjected for a period of 72 h to increasing concentrations of the drug: 10, 15, 20 and 25 nM. Cells were split 1/3 after each round of treatment and compared to a control in passage 13 for expression of CD133. Images of parental JoPaca-1 and sub clones in culture were taken on an inverted microscope (DM IRBE, Leica Microsystems, Wetzlar, Germany) equipped with a CCD camera (Progressive Scan CV-M1, JAI, Frankfurt, Germany). Scale bars were added in ImageJ (NIH, Bethesda, MD, USA) by calculating measured pixel sizes.

### Cytogenetics – multicolour fluorescence *in situ* hybridization (mFISH)

JoPaca-1 cells were treated with 10 ng/ml vinblastin at 37°C, 5% CO_2_ for 4 h to arrest them in metaphase. Metaphase spreads were prepared according to standard protocols [31]. In short, suspended cells were treated with 75 mM KCl for 20 min at 37°C and fixed with ice cold Carnoy's solution (methanol∶acetic acid = 3∶1) in three washing steps. The solution of fixed cells was dropped from 1 meter onto a tilted slide that had previously been wetted with 70% ethanol. mFISH was performed using the multi-colour probe kit 24XCyte (Metasystems, Altlussheim, Germany) according to the manufacturer's instructions. Hybridized metaphase spreads were analyzed using a computer-assisted fluorescence microscope (Axioplan 2, Zeiss, Göttingen, Germany) equipped with appropriate filter sets. Images were recorded with a CCD camera (Photometrics, Tucson, AZ, USA) and analyzed using Quips SpectraVysion™ Software (Vysis, now distributed through Applied Imaging).

### Clonogenic potential and tumourgeneity

#### *In vitro*-limiting dilution assay

JoPaca-1 cells were seeded in a 96-well plate in a one-to-one dilution series starting at 125 cells per well, each dilution in 16 replicates. The medium was not changed to enable autocrine signalling. After 10 days, wells showing distinct colonies were counted. After 15 days, colonies showing diverse morphological phenotypes were photographed.

#### *In vivo*-limiting dilution assay

Initial xenograft experiments were performed by injecting 1 Mio cells in NOD.Cg-Prkdcscid Il2rgtm1Wjl (NOD/SCID/γ or NSG) mice. To assess in vivo tumorigenicity of JoPaca-1, 104, 103 and 102 cells, respectively, were injected in 70 µl Matrigel (2 mg/ml) (BD, Heidelberg, Germany) into the pancreatic body of NSG mice. Successful engraftment of tumours and subsequent growth was monitored by regular palpation of the implantation site.

To quantify clonogenic potential and tumour-initiating frequency, data were analysed by a regression-fit algorithm (ELDA) [32].

### H&E staining and immunohistochemistry of xenografts and primary tumours

Explanted tumours were embedded in paraffin blocks and sections were cut at 4 µm thickness using a microtome. Haematoxilin and Eosin (H&E) staining as well as immunohistochemistry (IHC) were done according to standard procedures. Briefly, tissue samples were deparaffinised using Roticlear (A538, Carl Roth, Karlsruhe, Germany) and hydrated in a graded ethanol series. Antigen retrieval was achieved by heating in 10 mM citrate buffer (pH 6.1). Slides were blocked using Power Block (Biogenex, Fremont, CA, USA) and incubated with the primary antibody: anti-ALDH1 (Mat. No. 611194, BD Biosciences) used at 1∶1000; anti-cytokeratin 19 used at 1∶50; anti-cytokeratin AE1/AE3 (code M3515, DakoCytomation) used at 1∶300; anti-CD133/1 (AC133) (130-090-422, Miltenyi Biotech) used at 1∶50; and a universal negative control containing mouse IgGs (IS750, DakoCytomation) used at 1∶2 dilution. The slides were incubated at 4°C for 16 h. For detection of the primary antibody, a secondary anti-mouse antibody conjugated with peroxidase (K4001, DakoCytomation) was applied according to the manufacturer's instructions. The colour reaction with diaminobenzodine (DAB) was counterstained with haematoxylin. Finally, slides were viewed using an Axioplan 2 microscope and images taken by an AxioCam HRc CCD camera (Carl Zeiss MicroImaging, Jena, Germany). Scale bars were added in the accompanying AxioVision 4 software.

### Immunofluorescence

For immunofluorescence of mesothelin and cytokeratin 19, JoPaca-1, BxPC-3 and HPDE c7 cells were grown in culture slides (Cat. 354559, BD Biosciences), fixed with 2% paraformaldehyde and permeabilized with 0.2% Triton X-100 for 5 min. Slides were blocked in phosphate buffered saline (PBS) with 10% bovine serum albumin (BSA) and epitopes retrieved with PBS supplemented with 10% proteinase K. IgG1 antibodies against mesothelin K1 (Cat. sc-33672, Santa Cruz Biotechnology, Heidelberg, Germany) and cytokeratin 19 (clone RCK108, Code-Nr. M 0888, DakoCytomation, Hamburg, Germany) as well mouse IgG1 (MCA928, Serotec, Puchheim, Germany) as a negative control were used at a dilution of 1∶50. The secondary antibody Alexa Fluor 488 goat anti-mouse IgG1 (A-11001, Life Technologies) was used at a dilution of 1∶500. Slides were mounted with aqueous medium containing 4′,6-diamidin-2-phenylindol (DAPI) to counter-stain nuclei (sc-24941, Santa Cruz Biotechnology). Images were taken on a wide-field microscope equipped with an AxioCam CCD camera (Zeiss Cell Observer, Carl Zeiss MicroImaging, Jena, Germany).

### Fluorescent assisted cell sorting (FACS)

Expression of CD133 was detected by FACS (FACS Canto II, BD Biosciences, Heidelberg, Germany) using phycoerythrin conjugated CD133/2 (293C3) and CD133/1 (AC133) antibodies (Milteny Biotech, Bergisch Gladbach, Germany). Activity of ALDH1 was measured using Aldefluor substrate +/− DEAB inhibitor (Stemcell Technologies, Grenoble, France). ALDH-bright cells (ALDH_br_) were detected in the FITC channel and compensated against phycoerythrin and vice versa using individually labelled cells as controls. Expression of the triplet CD44/CD24/ESA was tested using the following antibodies from BD Biosciences: EpCAM (ESA)-APC (Cat. 347200), CD44-PE-Cy7 (Cat. 560533), CD24-FITC (Cat. 555427). Incubation of cells with antibodies and substrates, FcR blocking and washing steps were done according to the manufacturer's instructions.

### Whole genome sequencing

Genomic DNA of the cell lines JoPaca-1 (passage 8) and BxPC-3 was extracted using standard protocols. Whole genome sequencing and read alignment was done using an Illumina HighSeq 2000 sequencer at the Centre National de Génotypage (Evry, France). Data of the top 17 non-synonymous coding genes mutated in pancreatic cancer was obtained from the Sanger Institute catalogue of somatic mutations in cancer web site (http://www.sanger.ac.uk/cosmic) [33]. In addition, three genes were included from the OMIM database under the search term “pancreatic cancer” (http://www.omim.org/entry/260350) [34]. All of these genes were checked for exonic non-synonymous mutations in BxPC-3 and JoPaca-1 (Table S2). Furthermore, non-coding variations associated with pancreatic cancer were obtained from genome wide association studies (GWAS) (Table S3). The enumeration of mutated and wildtype reads was done by hand using the ensembl genome browser [35] and integrative genome viewer [36] counting every mapped read.

### Sanger sequencing of *KRAS*

The region around codon 12 of *KRAS* was amplified from cDNA generated from the cell lines by standard PCR using Phusion polymerase (F-530, Fermentas, St. Leon-Rot, Germany) and the following primers: KRAS-F (5′-CCC CGC CAT TTC GGA CTG GG-3′) and KRAS-R (5′-ACT CCT CTT GAC CTG CTG TGT CG-3′). The PCR products were sequenced by GATC (Constance, Germany) using primer KRAS-F.

## Results

### JoPaca-1 cells derive from poorly differentiated ductal adenocarcinoma of the pancreas

For the isolation of primary tumour cells, fresh tissue was obtained from a 46-year old male patient suffering of pancreatic cancer, who underwent a pylorus-preserving pancreatecto-duodenectomy (pp-Whipple operation). He died five months after surgery. The histopathological examination of the tissue revealed a poorly differentiated ductal adenocarcinoma of the pancreas, with tumour infiltration of the duodenum, the intrapancreatic bile duct and the peripancreatic soft tissue. Perineural, lymphogenic and hematogenic tumour infiltration could be found as well as two lymph node metastases in 22 regional lymph nodes. The TNM stage was pT3, pN1 (2/22), G3. Clinically, there was no occurrence of distant organ metastases. The primary cell line JoPaca-1 was isolated from the resected cancer tissue as described in detail in the methods section. The resulting population of pure tumour cells was frozen after four growth passages. For the experiments reported below, cells of 6 to 19 passages were used. The cells were grown in standard cell culture medium IMDM supplemented with 10% FCS and 1% PS.

### Subclones of JoPaca-1 reveal phenotypical heterogeneity of the parental population

**Figure 1:**
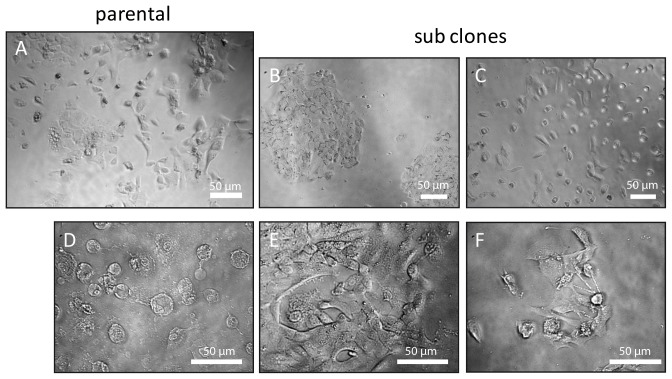
Growth behaviour and cell morphology. Phase contrast images are presented of JoPaca-1 parental cells and subclones showing different growth characteristics and morphologies. A. JoPaca-1 parental cell population. B,C. Two different growth characteristics can be observed: close-contact island formation (B) and no-contact spreading (C). D–F. Three different cell shapes were observed: round and vesicular (D), spindle-like (E), and pseudo-pod forming (F) cells.

Single cells of JoPaca-1 grown in complete isolation gave rise to colonies that exhibited a varying morphology (Fig. 1). Typically, two forms of growth could be observed: close contact island formation and no-contact spreading (Fig. 1B, C). Cellular heterogeneity was also reflected by the range of different cell shapes that were observed. Round and vesicular cells occurred as did a spindle-like phenotype. Also, the formation of long pseudopods could be seen (Fig. 1D–F). Both, different growth characteristics and the various cell shapes could also be detected in the parental population (Fig. 1A) indicating its good representation by the isolated sub clones.

### JoPaca-1 cells are tetra- to pentaploid and genomically instable

**Figure 2:**
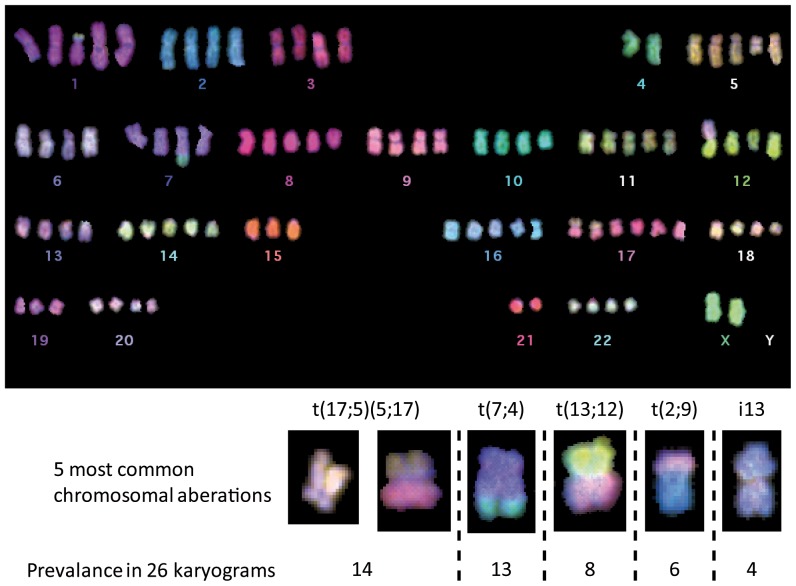
Karyogram and common chromosomal aberrations. A representative karyogram of JoPaca-1 is shown here. JoPaca-1 is a tetra- to penta-ploidic cell line. Below the overview picture, the five most commonly observed aberrations in 26 karyograms are presented. Translocations could be identified by hybridization of differently coloured probes resulting in dual-coloured chromosomes. Inversion of chromosome 13 (i13) is the fusion of two q-arms at the kinetochore. The Y-chromosome is often lost in tumour cells as it is in this representation.

JoPaca-1 was arrested in metaphase to study the karyotype and chromosomal aberrations. Metaphase spreads of 26 cells were analysed. They displayed a tetra- to pentaploidic karyotype with five commonly observed chromosomal aberrations (Fig. 2). These are the translocations t(17;5)(5;17), t(7;4), t(13;12), and t(2;9) and an inversion of chromosome 13 (i13). In total, 47 different aberrations were observed in the 26 karyograms. But for the relatively frequent aberrations of above, most (38) were observed only once. Three other translocations were found twice or three times (Table S1). The Y-chromosome was lost in 20 out of 26 karyograms.

### JoPaca-1 shows increased resistance to gemcitabine over BxPC-3

**Figure 3:**
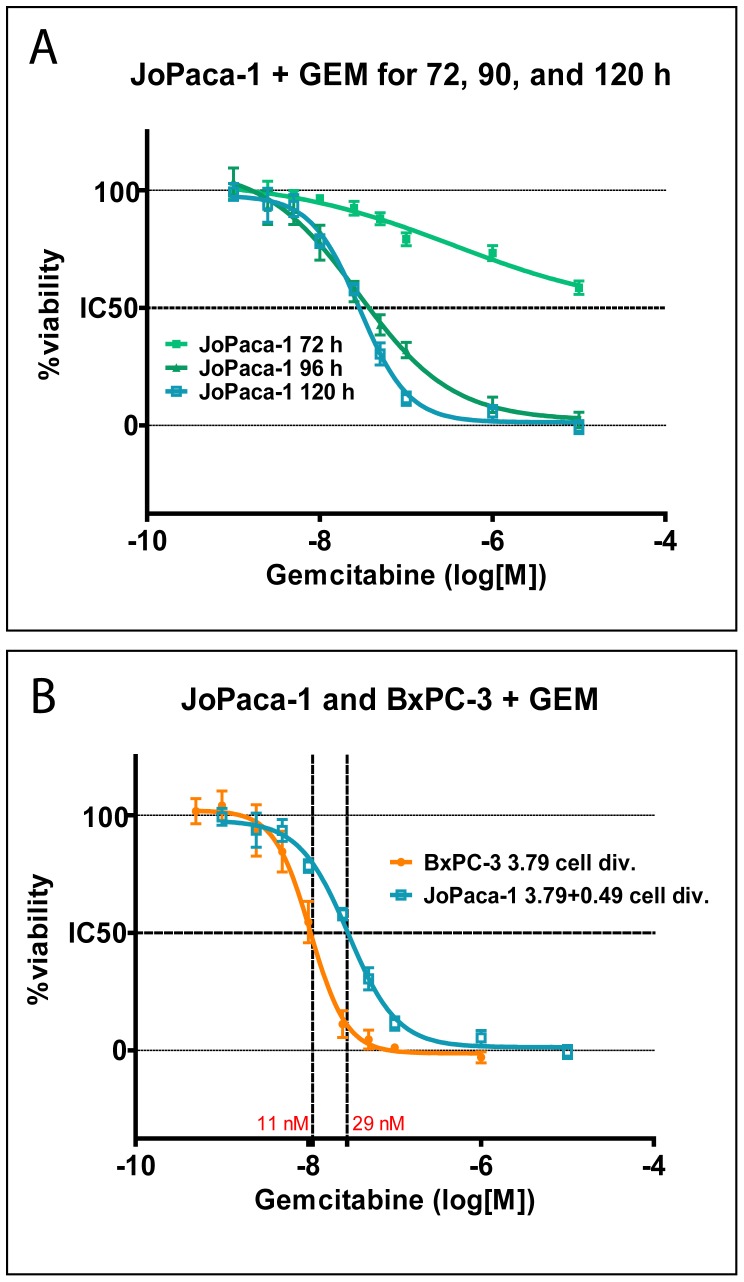
Response to gemcitabine dependent on time and growth rate. BxPC-3 and JoPaca-1 cells we treated with increasing concentrations of gemcitabine for 72, 96, and 120 hours. Cell viability was normalized to the non-treated controls. Data points represent averaged results of six replicate experiments with standard deviations indicated by error bars. A. Increasing incubation time with gemcitabine results in increased cytotoxicity for JoPaca-1. B. Cell viability was compared between 3.79 and 4.28 cell divisions for BxPC-3 and JoPaca-1, respectively. JoPaca-1 shows a higher tolerance towards gemcitabine than BxPC-3 even after extended incubation of 0.49 cell divisions.

JoPaca-1 cells were compared to BxPC-3 in their response to gemcitabine, the standard first-line chemotherapeutic treatment of pancreatic cancer. In absence of the drug, the doubling time was 28 h for JoPaca-1 and 19 h for BxPC-3. During a 72 h growth period, this corresponds to 3.79 cell divisions of BxPC-3 and 2.57 of JoPaca-1. In order to divide 3.79 times, JoPaca-1 would require 106 h. Consequently, a prolonged incubation with gemcitabine had a stronger effect on cell viability of JoPaca-1 (Fig. 3A). Compared to BxPC-3, JoPaca-1 showed a substantially higher tolerance to gemcitabine with an IC_50_ of 28 nM compared to 11 nM for BxPC-3 even after half of the cell population underwent an additional round of mitosis (3.79+0.49 = 4.28 cell divisions = 120 h) (Fig. 3B). Therefore, increased resistance of JoPaca-1 is independent of the rate of proliferation.

### JoPaca-1 cells are highly tumorigenic and have increased clonogenic potential

**Figure 4:**
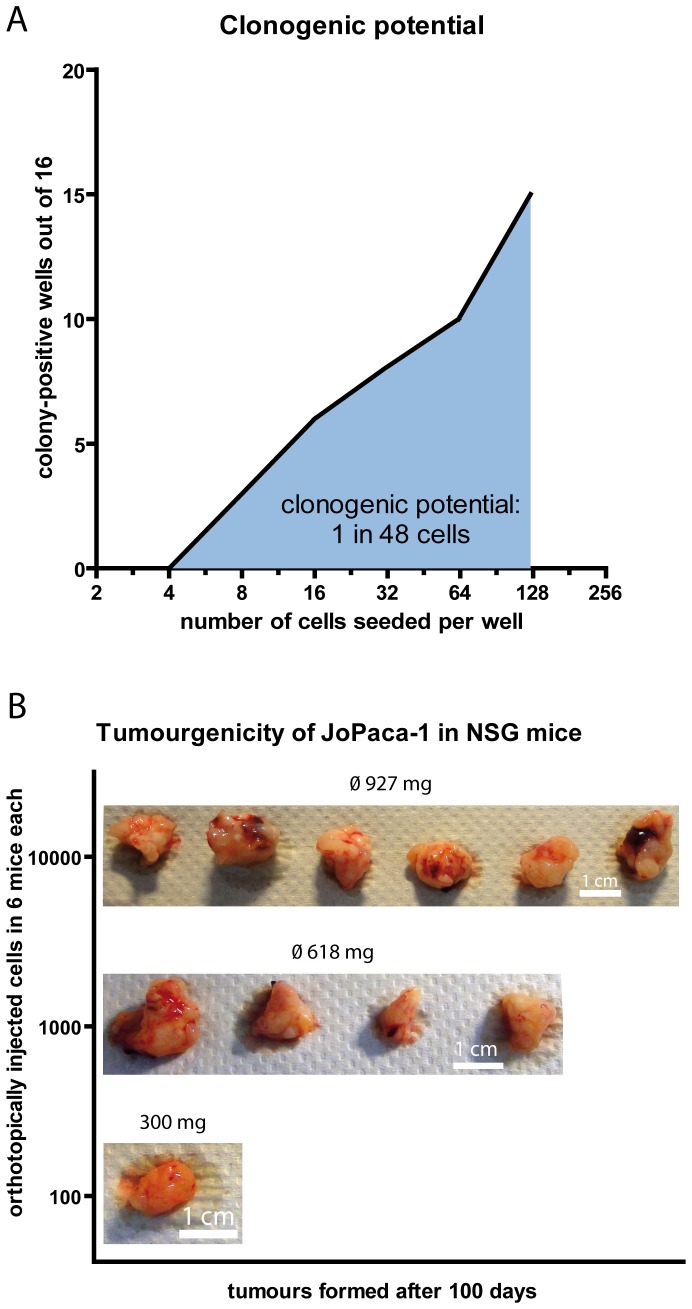
Clonogenic potential and tumourgenicity. Clonogenic potential and tumourgenicity of JoPaca-1 were determined by limiting dilution assays *in vitro* and *in vivo*. A. Dilution of cells in a 96-well plate and counting of colony-containing cells after 10 days lead to a clonogenic potential of 1 cell in 48 as calculated by ELDA [32]. B. Orthotopic injection of 10.000, 1.000, and 100 JoPaca-1 cells into 6 NOD.Cg-*Prkdc^scid^ Il2rg^tm1Wjl^* mice each resulted in formation of tumours which were excised, weighted and photographed. Average tumour masses are shown.

To establish clonogenic potential of JoPaca-1, cells in passage 10 were counted and seeded in a 1 to 1 dilution series in two 96-well plates starting from 125 cells per well down to 4 cells per well. Each dilution was done 16 times. After 15 days of incubation, wells containing distinct colonies were counted. Computational analysis of colony counts using the “Extreme LDA” algorithm [32] showed that one cell in 48 (∼2%) was able to form a colony (Fig. 4A). This clonogenic potential is ten times higher than that of the established cell line Capan-1 with 0.2% [19].

To establish the tumourgenicity of JoPaca-1 *in vivo*, three groups of six immune compromised NOD.Cg-*Prkdc^scid^ Il2rg^tm1Wjl^* mice were injected orthotopically with 10^4^, 10^3^, and 10^2^ cells, respectively. After 100 days, all mice were still alive. In total, 11 mice had formed tumours: 6/6 for the animals with 10^4^ cells, 4/6 with 10^3^ cells and 1/6 with 10^2^ cells (Fig. 4B). Tumour-initiating frequency was estimated by ELDA algorithm at 1 cell in 831 with a lower and upper limit of 1 in 2114 and 1 in 327, respectively. The tumours were excised and weighted. The tumour weight correlates directly with the number of injected cells. With 10^4^ cells, the average tumour weight was 927 mg; induction with 10^3^ cells gave rise to tumours of 618 mg on average. The single tumour resulting from 10^2^ cells had 300 mg.

### JoPaca-1 cells invade surrounding normal tissue and show metastasizing potential in vivo

**Figure 5:**
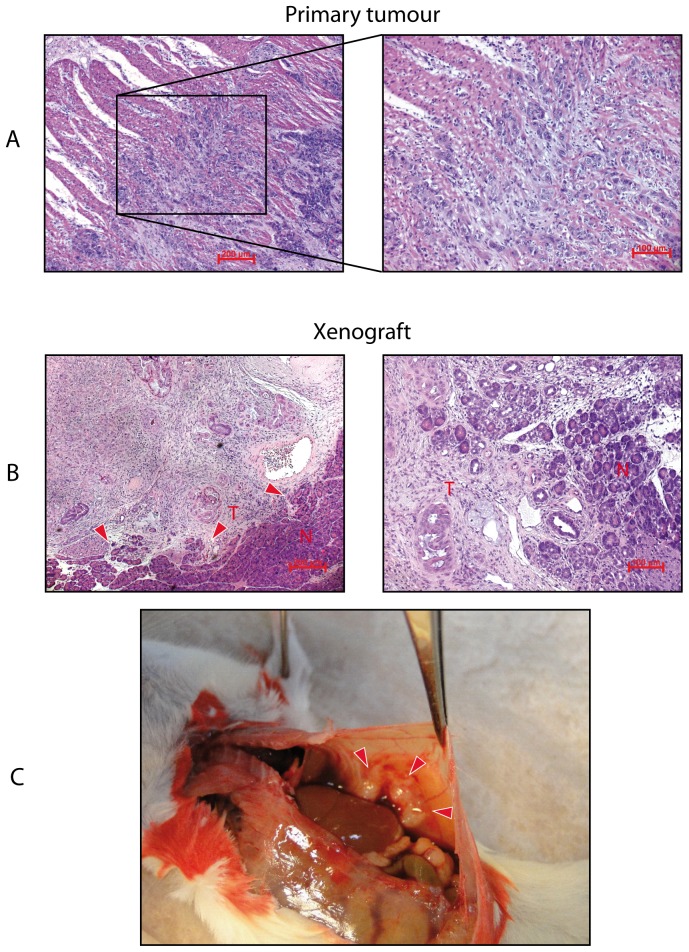
Micro- and macroenvironment of primary and xenograft tumours. Shown here are H&E stains of primary (A) and xenograft (B) tissue slices. A. Smooth muscle tissue of the duodenum was infiltrated by tumour cells of the primary tumour. B. Invasive front of xenograft tumours with areas of infiltrative tumour expansion into the adjacent normal pancreas tissue (arrow heads). C. One out of three NSG mice developed metastasis during initial experiments when 1 Mio cells were injected orthotopically (arrow heads).

The primary tumour had invaded smooth muscle tissue of the proximal duodenum (Fig. 5A). Xenograft tumours were not capsulated; moreover they revealed an invasive front with areas of infiltrative tumour expansion into the adjacent normal pancreas tissue (Fig. 5B) Metastases could be observed in one out of three mice during initial experiments but were not analyzed further (Fig. 5C).

### Primary and xenograft tumours raised from JoPaca-1 have very similar histology

**Figure 6:**
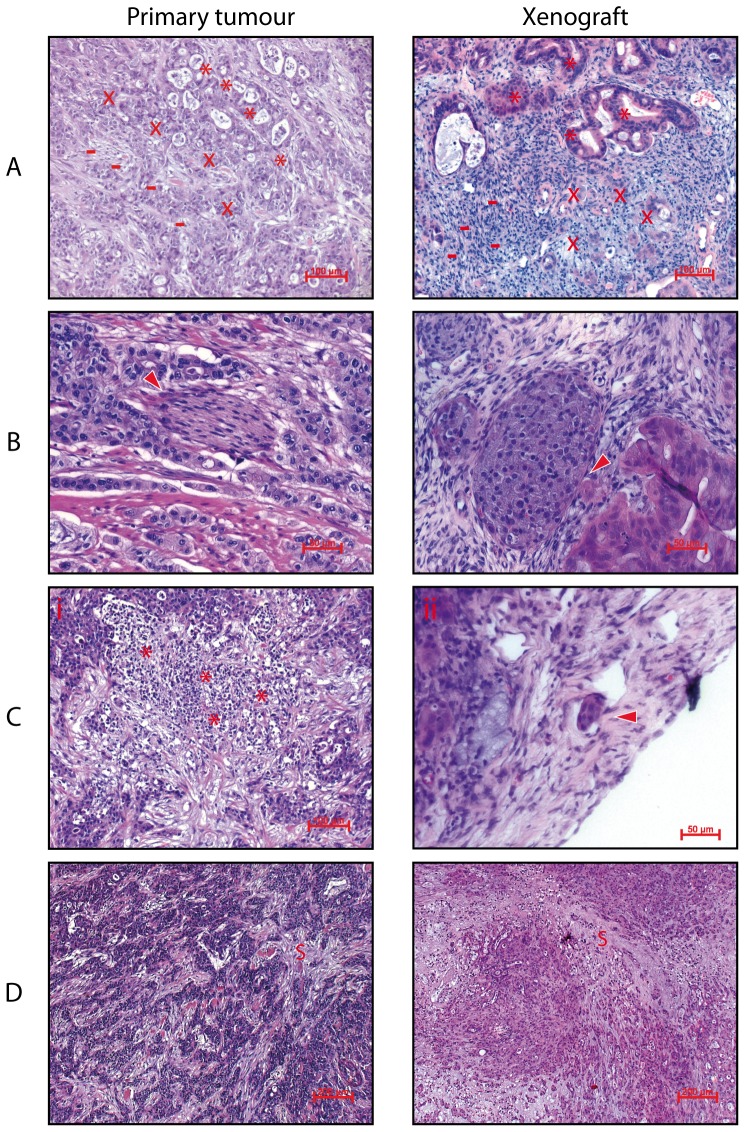
Histological comparison of primary and xenograft tumours. Shown here are H&E stains of primary and xenograft tumours. A. Primary and xenograft tumours show similar differentiation phenotypes ranging from overall poor differentiation (−) over micropapillary growth (x) to more differentiated ductular formations (*). B. Perineuronal invasion can be observed in both primary and xenograft tumours (arrow heads). C. Shown here are areas of tumour necrosis, demarcated by neutrophil granulocytes (C i, asterisks) and tumour infiltration of lymphatic vessel (C ii, arrow head). D. Distribution of stroma (S) in primary and xenograft tumour tissue. Tumour cells of both specimens are embedded in desmoplastic tumour stroma. While stroma in the primary tumour is dispersed throughout the tumour, it is more localized in the xenograft.

Histopathologically, the xenograft tumours show a high degree of similarity to the primary tumour. Cellular morphology and growth patterns were overall poorly differentiated characterized by single cellular or solid growth. However, areas of micropapillary growth patterns and ductular formations were found in both primary and xenograft tissue (Fig. 6A). Additionally, migratory and invasive behaviour was observed to a similar degree. Primary and xenograft tissues showed perineural infiltration and invasion into lymphoid and blood vessels (Fig. 6B, Cii and 11C).

Features owing to the immunological characteristics were naturally more pronounced in the primary tumour than in the xenograft as NSG mice are incapable of mounting an innate or adaptive immune response. These immunological features include areas of tumour necrosis that were demarcated by neutrophil granulocytes (Fig. 6Ci) as well as infiltrating inflammatory cells, mainly lymphocytes and granulocytes. In the primary tumour, cells are embedded in desmoplastic tumour stroma. Xenograft tumours are partly interspersed with stromal tissue as well. Here, these areas are localized and not as omnipresent as in the primary tumour (Fig. 6D).

### JoPaca-1 expresses cytokeratins and the tumour marker mesothelin

**Figure 7:**
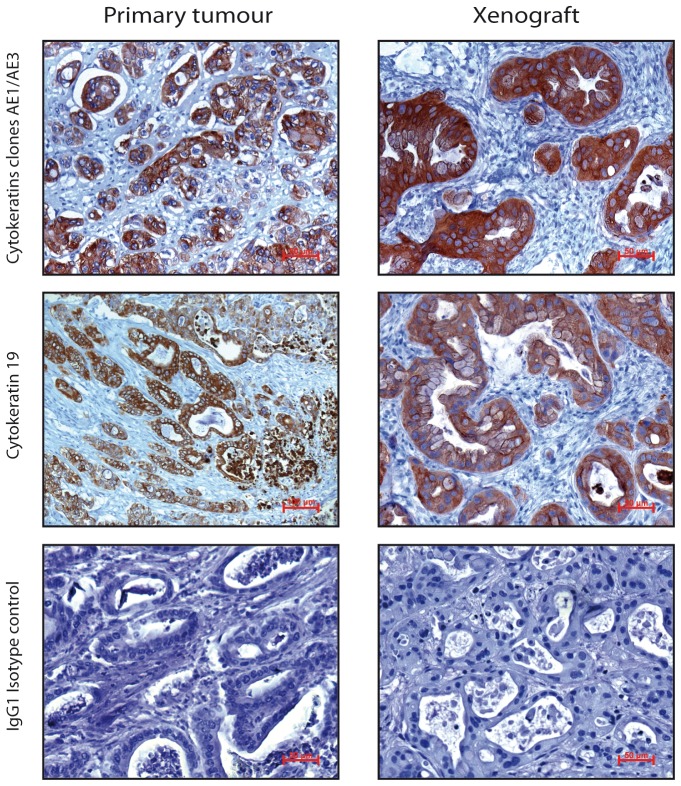
Immunohistochemistry of cytokeratins. Immunohistochemistry was performed on primary and xenograft tumour slices. Cytokeratins AE1/AE3 and more specifically cytokeratin 19 were expressed in ductal structures of primary and xenograft tumours (brown). Isotype controls were negative.

**Figure 8:**
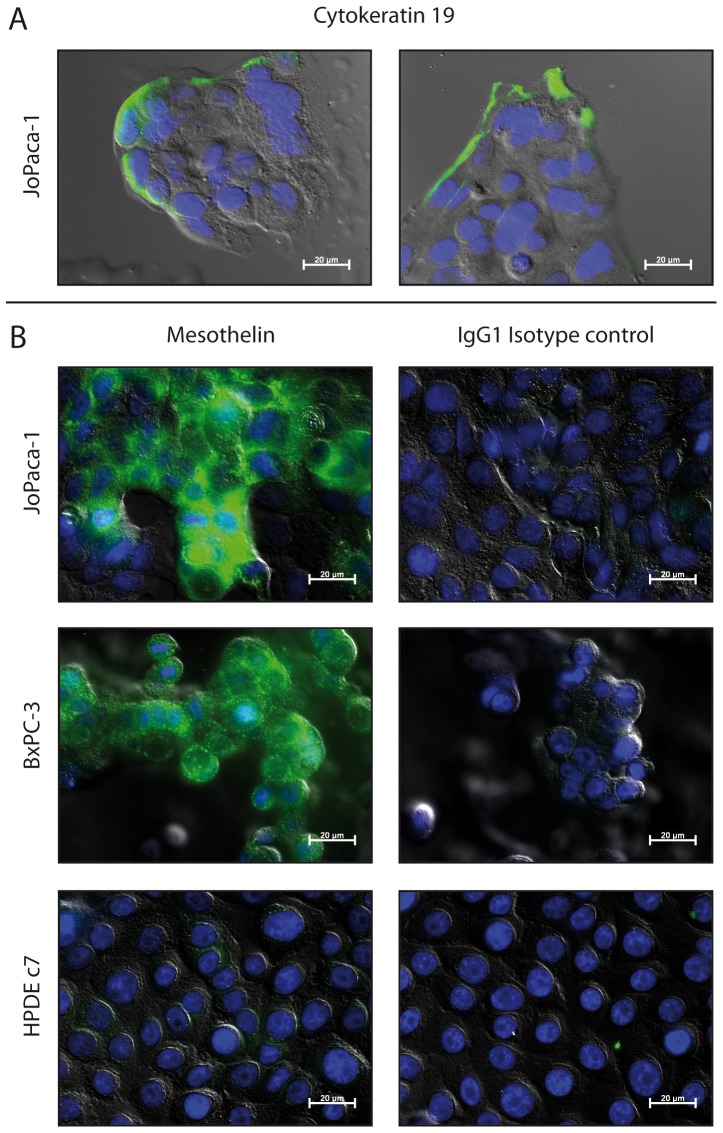
Immunofluorescence of cytokeratin 19 and the tumour marker mesothelin in cell culture. Phase contrast images are presented that show mesothelin and cytokeratin 19 as detected in fixed JoPaca-1, BxPC-3 and HPDE c7 cells. Both proteins were marked with primary and FITC-conjugated secondary antibody (green). Nuclei were stained with DAPI (blue). Isotype controls were negative. A. Cytokeratin 19 is expressed at the growing front of JoPaca-1 cells. B. JoPaca-1 and the established cell line BxPC-3 both express mesothelin while the normal pancreatic ductal cell line HPDE c7 does not.

Immunohistochemistry of cytokeratins was performed on primary and xenograft tumour tissue slices. Both, primary and xenograft tumours stained positive for the pan-cytokeratin clones AE1 and AE3. Expression of cytokeratins in the primary tumour was slightly more dispersed than in the xenografts, where it clearly stained the epithelial cells lining the luminal side of the ducts. More specifically, expression of cytokeratin 19 was even more characteristically ductal in both tissues. Isotype controls were negative (Fig. 7). Immunofluorescence confirms expression of cytokeratin 19 in isolated JoPaca-1 cells. Cytokeratin 19 is an intermediate filament protein and was expressed by JoPaca-1 at the growing front of a cell colony (Fig. 8A). Additionally, JoPaca-1 was tested for expression of the tumour marker mesothelin. The established pancreatic cancer cell line BxPC-3, and the normal pancreatic ductal cell line HPDE c7 were used as positive and negative controls, respectively. Expression of mesothelin could be detected in JoPaca-1 and BxPC-3 cells, but not in the normal pancreatic duct cells HPDE c7. Isotype-controls were negative (Fig. 8B).

### JoPaca-1 cells are highly enriched in cancer stem cell markers

**Figure 9:**
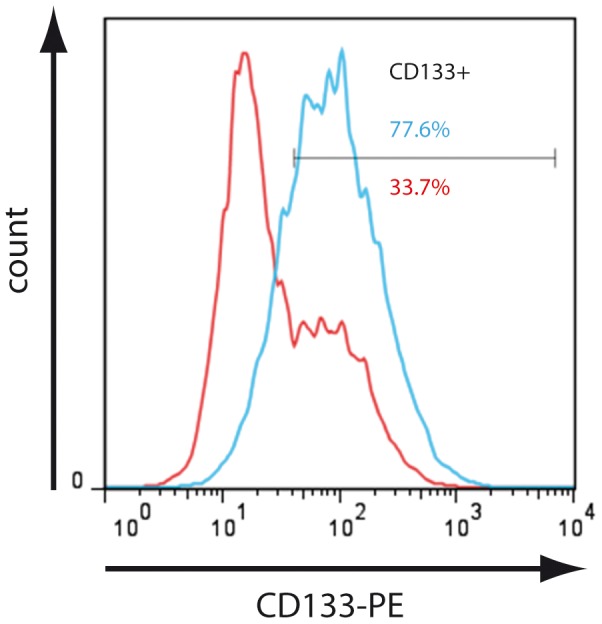
Enrichment of CD133 upon gemcitabine treatment. The CD133+ content of JoPaca-1 cells was determined via FACS after long-term treatment with gemcitabine (blue) and in comparison to a non-treated control (red). Prolonged gemcitabine exposure led to an increase of the CD133+ population from 33.7% to 77.6%.

**Figure 10:**
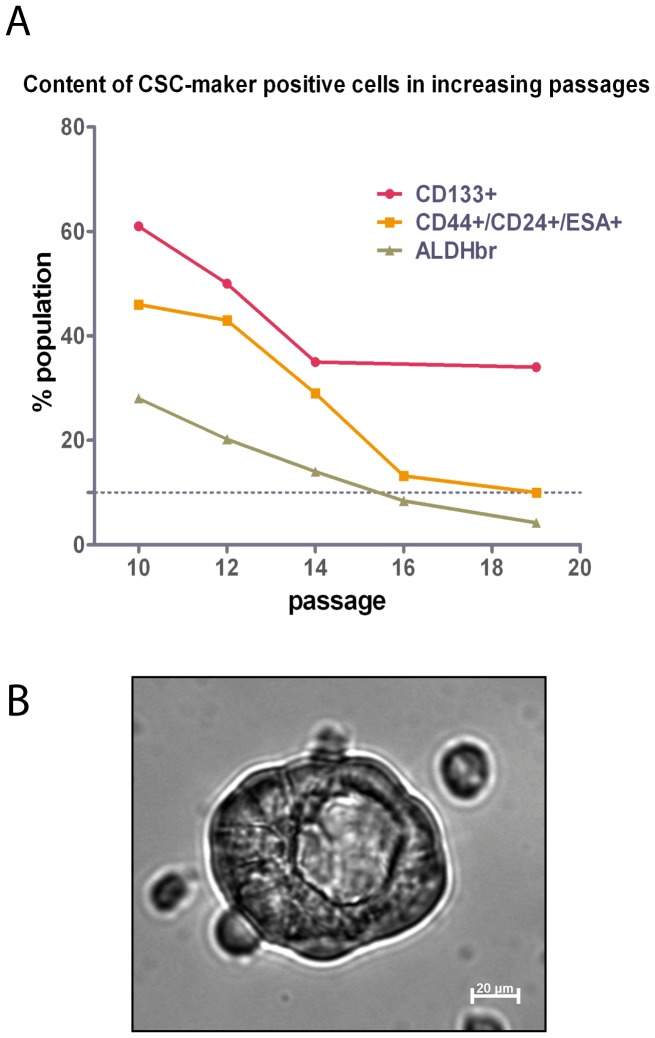
Content of CSC-marker positive cells in increasing passages and a hollow tumoursphere. A. Shown here is the content of cancer stem cell marker positive cells in increasing passages. Three different markers were determined by FACS for each passage: CD133+, CD44+/CD24+/ESA+ and ALDH bright (ALDHbr). Each data point represents the percentage of marker positive cells of the total population of JoPaca-1. B. Phase contrast image of a hollow tumoursphere of JoPaca-1 after cultivation in dedicated medium in a low-attachment culture plate.

Self-renewing cancer stem cells are thought to be the driving force behind tumour progression and responsible for resistance against chemotherapy. To identify pancreatic cancer stem cells *in vitro*, several markers have been used to single out sub-populations with an increased potential to initiate tumour formation in immune compromised mice. Among these are the triplet CD44+/CD24+/ESA+ [14], CD133+ [23], and more recently the activity of aldehyde dehydrogenase 1 (ALDH1) [18]. In JoPaca-1, all sub-populations were present individually or in a partly overlapping manner as summarized in Table 1 (the relevant FACS data are shown in Fig. S1). CD133+ cells were especially abundant in early passages (P6-P12). However, their abundance decreased with higher passage numbers until a steady level of about 35% was reached at passages 14 to 19 (Fig. S2). CD133-content of other pancreatic cancer cell lines, determined under the same conditions, was found to be much lower as summarized in Table 2; related FACS data are shown in Figure S3. An escalating dosage of gemcitabine over 12 days enriched the CD133+ population of JoPaca-1 to up to 77% (Fig. 9). CD133 was expressed by about one third of JoPaca-1 cells at passage number 19; similarly 28 percent showed a high activity of ALDH1 (ALDH_br_). Looking at the overlap of both populations, 60% of CD133+ cells had high ALDH1 activity, whereas three quarters of the ALDH_br_ cells were also CD133+. The fraction of JoPaca-1 with high ALDH1 activity and expression of CD133 was 21% (Fig. S1B). Similar to the content of CD133+ cells, the contents of ALDHbr and CD44+/CD24+/ESA+ decreased with higher passages. While the population of CD44+/CD24+/ESA+ cells levels at about 10% in passages 16 to 19, activity of ALDH1 decreases below 10% at passage 16 and further to 4.8% at passage 19 (Fig. 10A).

**Table 1:** CD133+ and overlap with CD44+/CD24+/ESA+ and ALDHbr.

population	passage	percent
CD133+	6	80
	7	69
	10	61
	12	50
	14	35
	19	34
CD133+/CD44+/CD24+/ESA+	9	9
CD133+/ALDH_br_	10	21

Summary of cancer stem cell marker subpopulations in JoPaca-1 determined via FACS. CD133 expression was assessed over passages 6 to 19. Co-populations of CD133+ with CD44+/CD24+/ESA+ was determined at passage 9. Activity of aldehydedehydrogenase and its correlation with CD133+ is shown by the content of ALDH-bright (ALDHbr) cells in passage 10.

**Table 2:** Expression of CD133 in established pancreatic cancer cell lines.

cell line	CD133+ population [%]
AsPC-1	0.7
BxPC-3	0.71
Capan-1	0.33
FamPAC	0.27
MDAPanc-28	3.36
MiaPaCa-2	2.12

Listed here is the content of CD133+ populations in established pancreatic cancer cell lines AsPC-1, BxPC-3, Capan-1, FamPAC, MDAPanc28, and MiaPaCa-2 measured by FACS. Contour plots of the FACS analysis are shown in figure S3.

The ability to form tumour spheres in suspension is a hallmark of tumour initiating cells [23]. In low-adherences plates, cultivated in serum-free medium, 2.14% (+/−0.27%) of JoPaca-1 cells were able to form tumour spheres after two weeks. Morphologically, some of these tumour spheres were hollow and had an opening much like a blastula (Fig. 10B). This phenotype has been described before for spheroids that originate from pancreatic or other ductal tissues [37], [38], [39].

### ALDH1 is expressed in infiltrating JoPaca-1 cells

**Figure 11:**
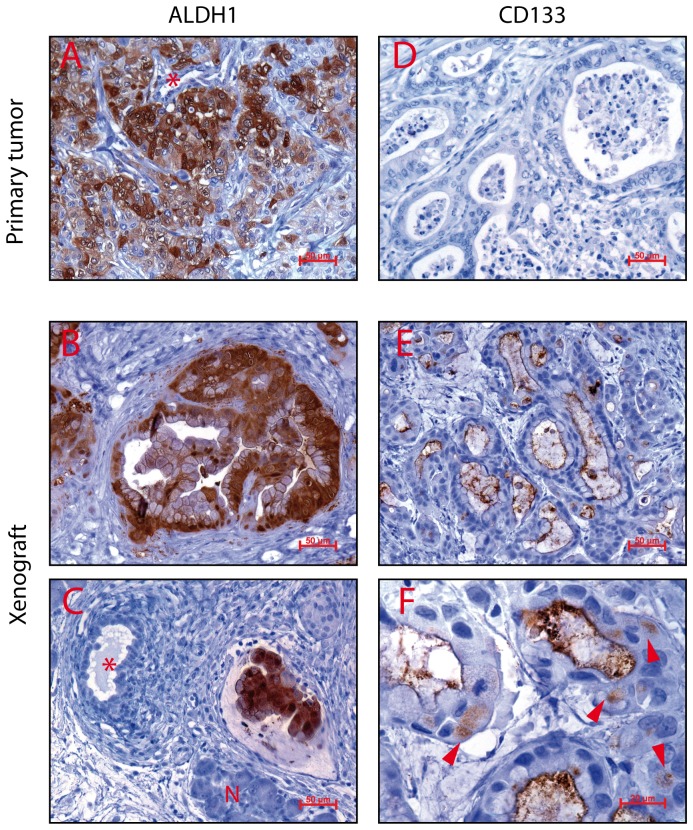
Expression of ALDH1 and CD133 in primary and xenograft tumours. Immunohistochemistry of ALDH1 and CD133 was performed for primary and xenograft tumours. A–C. ALDH1 expression was dispersed in primary (A) and more defined in xenograft tumours (B, C). Cells expressing ALDH1 were typically arranged in ductal structures with focal mucin inclusions (B). Ducts lined with cells not expressing ALDH1 lacked these inclusions (indicated by asterisks in A and C). Panel C shows ALDH1-positive cells invading a lymphatic vessel, while surrounding tumour cells were ALDH1-negative. Normal pancreatic tissue was present (Picture C, indicated by „N”). E,F. Expression of CD133 was observed only in xenograft tissues. Here, CD133 was expressed predominantly in the lumen of ducts. Ductal epithial cells stained positive at the apical membrane (E) and occasionally intracellularly (F, arrow heads). Detritus within the lumen was typically stained positive.

ALDH1 was expressed in both the primary tumour and xenograft tissues. The primary tissue shows a heterogeneous dispersal of expression, while staining was more defined in the xenograft tissues. Distinct clusters of ALDH1 positive cells could be observed in both tissues (Fig. 11). ALDH1-expressing cells organized in ductular-like complexes harboured large mucin inclusions while ducts with no ALDH1 expression did not. Figure 11C shows ALDH1 positive cells infiltrating a lymphatic vessel, while surrounding tumour cells are ALDH1-negative. CD133+ cells were stained in xenograft tissue slices at the apical membrane of ductal epithelial cells. Intracellular expression was detected in some cells. Cellular debris within the ductal lumen was strongly stained with CD133/1 (AC133) antibody. We could not detect CD133 in the primary tumour tissue (Fig. 11).

### JoPaca-1 is heterogeneously mutated at alleles characteristic of PDAC

Whole-genome sequencing was performed on both the primary cell line JoPaca-1 (passage 8) and BxPC-3 (passage 18). Both genomes were sequenced with a median 15-fold coverage. Paired-end sequencing was done with an average read length of 100 bases and a base call accuracy of 99.98% (average Phred score of 38). The sequence information is available from the Sequence Read Archive (SRA) under the accession number ERP001513 (http://www.ebi.ac.uk/ena/data/view/ERP001513). Non-synonymous coding mutations were found in 10 of the 20 most often mutated genes in pancreatic cancer, as listed in the cancer gene census [40] and the Online Mendelian Inheritance of Man database (www.omim.org). For each location, the number of mutated and wildtype reads was counted and normalized to the total number of reads in that locus. Amino acid changes were listed and positional information included in case of genes with one transcript only. In BxPC-3, all analyzed genes except *MLL3* and *SMARCA4* are either purely wildtype or mutated with all reads containing either the mutated or wildtype base at that locus. JoPaca-1 on the other hand is more diverse in its mutational spectrum. Common mutations in *BRCA1* and *2*, *PALLD*, and *KRAS* are present only in parts of the cell population. Most genes are mutated only in 1 or 2 locations while *MLL3* is diversely mutated in many locations in both cell lines (Table S2).

To describe not only important parts of the exome, non-coding variations that have been found to be significantly associated with pancreatic ductal adenocarcinoma were selected from publically accessible results of genome wide association studies up to January 2012 (Table S3). Selection criteria were significance of association (p-value) and presence in either BxPC-3 or JoPaca-1. In the table, each original source is identified by the first three letters of the respective first author's surname: Innocenti [41], Low [42], Petersen [43], [44], Diergaarde [45], and Rizzato [46]. The p-value thresholds for each study were as follows: Innocenti: p≤10^−4^; Low: p≤10^−5^; Petersen/Diergaarde/Rizzato: p≤10^−2^. The column “associated genes” lists genes or loci associated to the variation based on proximity to the site of variation.

Sanger sequencing of three JoPaca-1 subclones isolated by limiting dilution showed codon 12 of *KRAS* to be mutated to G**T**T (Val). In the complete pool of JoPaca-1 cells, however, also the wildtype allele G**G**T (Gly) could be detected. BxPC-3 and HPDE c7 carry the wildtype G**G**T to 100%, while Capan-1 and FamPAC are mutated to G**A**T (Asp) and G**T**T (Val) respectively (Fig. S4).

## Discussion

Cancer research focuses more and more on targeted therapies rather than broadband chemotherapy. The evaluation of new, targeted drugs for cancer therapy usually starts with *in vitro* assays in cell culture. For working in the appropriate molecular context an extensively characterized model system is essential. Tumour tissues from different patients, even if taken from the same organ, are known to differ extensively in their phenotype and genotype [47]. These interindividual differences are also reflected by the strong variations between well established cell lines and include features like cell morphology, grade of differentiation, quality and quantity of chromosomal aberrations, mutational status as well as the content of tumour initiating cells or cells expressing cancer stem cell markers. In order to model this situation in preclinical experiments, it is important to use as many different established cell lines as possible. We recently studied the protein expression of 21 established pancreatic tumour cell lines [48], for example. While common features could be detected, unsurprisingly also very many individual expression variations were observed in this study. Furthermore, even intraindividual tumour cells derived from tumours of one and the same patient show a great variety, as is illustrated here. Within a heterogenic tumour cell population, chemotherapy often leads to a selection of a resistant subpopulation that eventually culminates in recurrence of the tumour as well as the formation of metastases. To model this intratumoural heterogenity most suitably, early passages of primary tumour cells can be utilised, thereby representing the complexity and heterogeneity of tumour tissues in a much better way.

We describe here the isolation and characterization of a novel primary pancreatic cancer cell line named JoPaca-1. The tumour cells were isolated directly from human tumour tissue of a poorly differentiated pancreatic adenocarcinoma. The pool of JoPaca-1 tumour cells contains cells of different stages of differentiation. This was maintained even after extended sub-cultivation *in vitro*; as shown at the phenotypic level by differing colony formation behaviour of undifferentiated cells growing as single cells and more differentiated cells growing in a cobblestone pattern that is characteristic for epithelial cells. Differences in cell morphology underlined this heterogeneity. In mouse xenografts, different stages of differentiation were observed. At the genotypic level, heterogeneity is reflected by 47 different aberrations, most of which occurring only once in 26 karyograms. This plenitude indicates a much higher number of unobserved aberrations. The five most commonly observed aberrations were t(17;5)(5;17), t(7;4), t(13;12), t(2;9), and (i13) and have, to best of our knowledge, not been reported in pancreatic cancer to date. In combination, these features demonstrate the genomic heterogeneity and chromosomal instability of JoPaca-1.

Pancreatic cancer is a very aggressive form of cancer, which is reflected by the short survival time after diagnosis and the high mortality rate. Also the donor of JoPaca-1 survived for only five month after diagnosis. Histopathological analysis of the primary tumour revealed the presence of necrosis and infiltration of blood and lymphoid vessels, duodenal smooth muscle tissue along with perineural invasion and showed the high aggressiveness of this particular tumour. A comparison between the primary tumour and xenograft tissues that arose from JoPaca-1 showed great similarity in terms of differentiation, invasive behaviour, and desmoplastic reaction, underlining how well the newly established JoPaca-1 cell line actually represents the primary tumour. In contrast to xenografts raised from conventional cell lines, which are often capsulated, non-invasive and overall less similar to primary PDAC, JoPaca-1 shows a much higher resemblance to the primary tumour.

To aid pathological characterization of pancreatic cancer, genes that typically exhibit strong expression in PDAC are used as tumour markers. Mesothelin is such a molecule. It is a glycosylphosphatidylinositol-anchored cell-surface protein and likely to be involved in cell adhesion. The protein is over-expressed in PDAC [49], [50] and used for pathological characterization of tumours [51]. Immunofluorescence confirmed the expression of mesothelin in both pancreatic cancer cell lines BxPC-3 and JoPaca-1, no expression was detected in the normal cell line HPDE c7, showing the tumour specific expression of mesothelin. Cytokeratin 19 (CK-19) is an intermediate filament protein and provides structural integrity especially for epithelial cells. It is expressed in many adenocarcinomas of the gastrointestinal tract [52] and used as a marker of differentiation. Interestingly, JoPaca-1 expresses CK-19 at the growing front of a tumour cell colony. This observation corresponds with the fact that intermediate filaments are reorganized as part of the cytoskeleton during cell migration [53].

To characterize *in vitro* proliferation of JoPaca-1, we determined the doubling time and its ability to form colonies from single cells. Knowledge of the doubling time of a cell line is especially important when comparing different cell lines and their response to chemotherapeutics that depend on mitosis. Therefore, response of BxPC-3 and JoPaca-1 to the antimetabolite gemcitabine was compared by doubling time and not mere incubation time. JoPaca-1 displayed a higher tolerance towards gemcitabine than BxPC-3. Even after 50% of JoPaca-1 had undergone an additional round of mitosis compared to BxPC-3, the IC50 was about 3 times higher than that of BxPC-3. This increased resistance could be related to the high content of cancer stem cells. Coherently, the population of stem cell marker positive (CD133+) cells was enriched after treatment with gemcitabine.

JoPaca-1 contains an exceptional number of putative cancer stem cells (CSCs) relative to other established cell lines. This could facilitate research on CSCs since usually only very small fractions of CSCs can be obtained from standard pancreatic cancer cell lines [23]. Most intriguingly, as few as 100 unsorted cells were able to form a tumour in one out of six immune deficient mice. Also the ability of single JoPaca-1 cells to grow in isolated wells and giving rise to colonies (clonogenic potential) is indicative of self-renewal and self-sustainment. Self-renewal and the ability to reconstitute tumours *in vivo* are hallmarks of cancer stem cells. The clonogenic potential was quantified *in vitro* and *in vivo* by limiting dilution assays and is relatively high with 2% (every 48^th^ cell) *in vitro* and 1/6 *in vivo* for as few as 100 injected cells (Fig. 4). For comparison, Rasheed *et al.* found that long established Capan-1 cells have a clonogenic potential of 0.2% *in vitro* and 10,000 xenografically enriched tumour cells were needed to give rise to tumours in 3 out of 17 cases (∼1/6) [19]. Thus, JoPaca-1 is 100 times more tumorigenic than xenografically enriched Capan-1 cells. They also showed that sorting for expression of ALDH1 or CD24/CD44 could increase clonogenic potential six-fold. ALDH1 expression and activity was confirmed in JoPaca-1 along with other cancer stem cell markers.

The use of molecular markers to identify cancer stem cells is still under debate [54], [55]. Since the discussion about the existence and role of cancer stem cells has been going on, many features have been suggested for identification. The current gold standard for validation of stem cell markers is an increased tumour-initiating potential in immune compromised mice [14], [19], [23]. Consequently, we analyzed JoPaca-1 for the most promising markers known to identify tumour-initiating cells in pancreatic cancer. Whether these markers are the most suitable ones for identification of CSCs is not under investigation here, as it would require more than one primary PDAC cell line in a larger study. We found that expression of CD133+, the triplet CD44+/CD24+/ESA+, and activity of ALDH1 in JoPaca-1 at passage 10 were much higher than reported in other cell lines, with 61%, 46%, and 28% respectively [54], [56], [57]. Even the overlap of two independent markers accounts for a substantial percentage of the whole JoPaca-1 population. However, the content of CD44+/CD24+/ESA+ and ALDHbr cells decreased after multiple passages *in vitro*. We therefore recommend using JoPaca-1 cells in low passage when focussing on cancer stem cell properties. Interestingly, when treated with increasing concentrations of gemcitabine, the CD133+ population recovered to almost the same level observed in passage 6. This confirms similar results obtained by Hermann *et al.*, who also showed that CD133+ pancreatic cancer cells were resistant to very high concentrations of gemcitabine (100 µg/ml) [23]. Very recently, expression of CD133 has been correlated with increased migration and invasion by epithelial to mesenchymal transition (EMT) [58]. The EMT potential of JoPaca-1 is currently under investigation. The high content of CD133+ cells in JoPaca-1 and the ability to reconstitute it by selection with gemcitabine facilitates large-scale functional studies focussed on this stem cell marker, such as RNAi screening or an *in vivo* analysis of drugs targeting cancer stem cells.

Expression of CD133 and ALDH1 was determined in the primary and xenograft tissues by immunohistochemistry. ALDH1 expression was often observed in infiltrating tumour cells, suggesting a correlation of ALDH1 expression and enhanced infiltrating properties of JoPaca-1 (Fig. 11C). This observation is in line with experiments performed by Rasheed *et al.*, who showed increased invasive, migratory, and metastatic behaviour of ALDH1 expressing cells [19]. The more defined expression in xenograft tissue suggests that correlating migration and invasion is more aggressive in the xenograft where the tumour has the opportunity to expand and is not restricted by a bulky tumour mass or an immune system as compared to the situation in the primary tumour. We could not detect CD133-expressing cells in the primary tumour tissue. Apparently, the particular slice of primary tumour material used in the analysis did not contain any CD133+ cells. We did, however, detect CD133- expressing tumour cells in the xenograft tumours, which were still in an infant stage compared to the primary tumour. Here, expression of CD133 was localized at the luminal side of ductal epithelia and in intraluminal cell detritus. Similar to previous observations [59], intracellular expression was rare and typically in cells close to ductal formations.

Whole genome sequencing of BxPC-3 and JoPaca-1 was carried out in order to investigate aberrations at the genomic level. This is, to our knowledge, the first time that pancreatic cancer cell lines have been sequenced entirely. Here, we only discuss part of the data and focus on important genetic variations in pancreatic cancer. Variations in protein coding regions were analyzed for the 17 most commonly mutated genes of pancreatic cancer. In both cell lines, we could detect 17 non-synonymous mutations resulting in an amino acid change. Functional consequences are already known for some of these proteins. Most notably, an activating mutation of the *KRAS* oncogene (G12V) is present in 89% of JoPaca-1, while BxPC-3 is a *KRAS*-wildtype cell line. *KRAS* G12D and G12V mutations are known to be driver mutations in the development of PanINs and preinvasive cancer, as was demonstrated in genetically engineered mouse models [60], [61]. We found that subclones of JoPaca-1 carried the G12V mutation (GTT allele) to 100%, suggesting that the pool is made up of cells that are either mutated (89%) or not (11%) (Fig. S4). In JoPaca-1, the tumour suppressor p53 is mutated only in codon 72 (100%) where a proline is exchanged for an arginine (P72R). This mutation lies in a proline-rich region and is one of the most investigated variations of p53. Functionally, it is important for growth suppression and apoptotic activity [62]. Clinical consequences of a p53 P72R mutation are known for head and neck cancer patients. They have a worse prognosis when treated with DNA damaging agents, which is attributed to an inhibition of the transcription regulator p73 by the 72R mutant p53 [63].

Mixed-lineage leukemia 3 (*MLL3*) is another gene frequently mutated in pancreatic cancer as well as colorectal [64] and breast cancer [65]. This gene encodes for a histone 3-lysine 4 methyltransferase and is part of the ASCOM complex involved in transcriptional coactivation and chromatin regulation. *MLL3* is known as a tumour suppressor and has been implicated as a transactivator of p53 within the ASCOM complex [66]. Five non-synonymous mutations were identified in both cell lines, BxPC-3 and JoPaca-1. Mutation rates were between 9% and 41%. *MLL3* is mutated in different locations to varying degrees, as opposed to p53 and *KRAS* in which mutation rates are higher and location specific.

In support of the mutator phenotype hypothesis [67], mutations in *MLL3* seem to be acquired at a later stage of tumour development (passenger mutations) and are not initiators of tumour formation as opposed to driver mutations like *KRAS* G12V/D [68]. Interestingly, we identified a mutation in *SMARCA4* in BxPC-3 and JoPaca-1 without an identification number. The protein is part of the SNF/SWI complex required for transcriptional activation by chromatin remodelling. Together with histone tail modification complexes like ASCOM, ATP-dependent complexes like SNF/SWI are the main contributors of nucleosomal remodelling for transcriptional regulation [69]. Palladin, a protein encoded by *PALLD*, is believed to change actin-organization by binding to actin-associated proteins, thereby influencing cytoskeletal dynamics [70]. One of these actin-associated proteins, ezrin, is overexpressed in pancreatic cancer and plays a role in invasion and metastasis [71]. Linkage of *PALLD* mutations to familial pancreatic cancers is controversial [72]. JoPaca-1 carries two mutations in *PALLD*, one of which is also present in BxPC-3. Mutations were detected in *BRCA1* and *2*, which are known to play an integral role in homologous recombination repair and are frequently mutated in breast, ovarian, and pancreatic cancer [73].

In addition to the non-synonymous coding mutations, we extracted detailed sequencing data of non-coding intergenic and intronic SNPs in JoPaca-1 and BxPC-3 associated with pancreatic cancer. Again, we only discuss the most significant ones. SNPs in the *CLPTM1L-TERT* locus (rs401681 and rs4635969) have been identified in several studies [42], [43], [44], [46] and are associated with multiple cancers [74], [75], [76], [77]. Both genes are known to be involved in carcinogenesis. *CLPTM1L* is associated with apoptosis and *TERT* encodes part of the telomerase [78]. rs4635969 is also located close to a microRNA (MIR4457) and could influence its regulation. Another SNP putatively conferring telomere dysfunction (rs708224) was discovered in a Japanese population of pancreatic cancer patients [42]. Both cell lines carry this intronic variant in the sequence of *BICD1* to 100 percent. The most significant association in this population was calculated for rs9502893, which might influence regulation of *FOXQ1* expression. This gene belongs to the forkhead box family of transcription factors. Down-regulation of another member of this family, *FOXM1* is known to be inhibiting proliferation, migration, and invasion of pancreatic cancer cells [79] while up-regulation favours an EMT phenotype [80]. Both cell lines are mutated in rs9502893. Future experiments have to show, whether *FOXQ1* plays a similar role as *FOXM1* and whether the associated T allele has a regulatory influence.

A strong association for pancreatic cancer risk was identified for rs4820599 [45]. In JoPaca-1, this location is mutated in 9 out of 32 reads (28%). The associated gene, *GGT1*, encodes the gamma-glutamyltransferase 1, which is involved in glutathione metabolism [45]. Significant associations of SNPs (rs630014, rs505922, and rs657152) within the first intron of the ABO gene and pancreatic cancer have been reported by Amundadottir and Petersen *et al.* and confirmed by *Rizzato et al.* [43], [46]. A frameshift mutation (G258del) in *ABO* determines the O-blood group. Interestingly, the protective T-allele at rs505922, that is present in JoPaca-1 but not in BxPC-3, is in complete linkage disequilibrium with the O-blood group. Previous studies have shown an increased risk for gastric and pancreatic cancer for individuals of the A and B blood groups [44].

Cancer is a genetic disease, thus the genomic sequence of cell lines is a valuable resource for basic research. The presented data on common somatic mutations and SNPs associated with pancreatic cancer in BxPC-3 and JoPaca-1 underline the heterogeneity of the two different cell lines and provide a deeper insight of the genetic make-up that is invaluable for the interpretation of future experiments with JoPaca-1 and future and past experiments with BxPC-3.

In conclusion, a wide selection of different cell lines exists due to the heterogeneity of carcinomas of the pancreas. Many of the established cell lines date back to the 1970s and they have been extensively used and characterized. This has the advantage that new results can be related to previous data on the same cell line. However, each such cell line represents only one subpopulation of cells of either the primary tumour or a metastasis. Therefore, new and well characterized cell lines are continuously needed to replenish the pool of suitable model systems. With the establishment and extensive characterization of JoPaca-1, a new valuable tool is added to the tool box of model systems that will contribute to the assessment of the mechanisms of the devastating disease that is pancreatic cancer. JoPaca-1 in passage 6 will be made publically available by submission of passage 4 to a cell line collection.

## Supporting Information

Figure S1 **Expression and overlap of CD44/CD24/ESA/CD133 in JoPaca-1.** A. Expression of putative stem cell markers CD44, CD24, ESA, and CD133 was determined by FACS using the respective antibodies conjugated to fluorescent dyes: Phycoerythrin-Cy7 to anti CD44, FITC to anti-CD24, APC to anti-ESA, and phycoerythrin to anti-CD133. Depicted here are two-dimensional dot plots showing population-overlap between ESA and CD44; ESA, CD44, and CD24 as well as ESA, CD44, CD24, and CD133. All events were normalized to the parental population (P2). B. Expression of CD133 and ALDH1-activity (ALDHbr) of JoPaca-1 cells were measured by FACS using phycoerythrin-conjugated CD133-antibody and the Aldefluor assay, respectively. Twenty one percent of JoPaca-1 cells were CD133+ and ALDH1-active.
(TIF)

Figure S2 **FACS histograms of CD133 content in JoPaca-1.** The content of CD133+ cells in JoPaca-1 was determined in increasing passages. Histograms are shown of cells stained with phycoerythrin-conjugated CD133-antibody (blue) and unstained cells (red) determined by FACS.
(TIF)

Figure S3 **FACS contour plots of cell lines with low CD133 content.** The CD133+ content of established pancreatic cell lines was measured by FACS. Depicted here are contour plots of unstained cells (red) and cells stained with phycoerythrin-conjugated CD133-antibody (blue). Outliers of the contour are shown as dots and were include in the analysis. The gate for CD133+/− discrimination was positioned close to the edge of unstained cells owing to the small fraction of CD133+ cells. To correct the resulting bias, fractions of positively gated unstained cells were considered as background and subtracted from the percentage of positively gated stained cells. Capan-1 and MDAPanc28 have close to no CD133+ cells while FamPAC and MiaPaCa-2 have a CD133+ content of about two to three percent.
(TIF)

Figure S4 **Sanger sequencing of** ***KRAS*** **codon 12.** Electropherograms are presented for codons 11, 12, and 13 of the *KRAS* locus of JoPaca-1 parental cells and three JoPaca-1 subclones as well as four established cell lines. Sanger sequencing of JoPaca-1 parental cells detected both mutated (dT) and wildtype (dG) basepairs at the second position of codon 12, whereas all JoPaca-1 subclones were mutated (dA). Established cell line BxPC-3 and the pancreatic normal cells HPDE c7 are of wildtype sequence while Capan-1 and FamPAC contain mutations dA (Asp = D) and dT (Val = V).
(TIF)

Table S1 **Chromosomal aberrations observed in the karyograms of 26 JoPaca-1 cells.** Listed here are all observed aberrations and their prevalence in 26 karyograms. “t” marks translocations and “i” stands for inversions.
(DOCX)

Table S2 **Most commonly observed mutations in pancreatic cancer listed for BxPC-3 and JoPaca-1.** Listed here are non-synonymous coding mutations of the top-17 mutated genes for pancreatic cancer (according to the COSMIC database) as well as *BRCA1/2*, *PALLD* and *PALB2* (OMIM database) for BxPC-3 and JoPaca-1. Mutated bases are shown beneath each cell line with the respective read counts in parentheses. The degree of mutation is given in percent. SMAD4 is largely deleted in BxPC-3 and JoPaca-1.
(DOCX)

Table S3 **Selected SNPs from genome wide association studies in pancreatic cancer listed for BxPC-3 and JoPaca-1.** The table lists significant non-coding variations for PDAC and their degree of mutation in the cell lines BxPC-3 and JoPaca-1. Mutations were selected from genome wide association studies up to January 2012. Sources can be identified by the first three letters of the first authors' last names: Innocenti [41], Low [42], Petersen [43], [44], Diergaarde [45], and Rizzato [46]. Mutated (mut) and wildtype (wt) bases are shown beneath each cell line with the respective read counts. The degree of mutation is given in percent.
(DOCX)
