# Supplementary figures and images for: Establishment and Characterization of a Highly Tumourigenic and Cancer Stem Cell Enriched Pancreatic Cancer Cell Line as a Well Defined Model System
Source: PLoS One. 2012 Nov 12;7(11):e48503. doi: 10.1371/journal.pone.0048503 (PMC3495919; doi:10.1371/journal.pone.0048503)

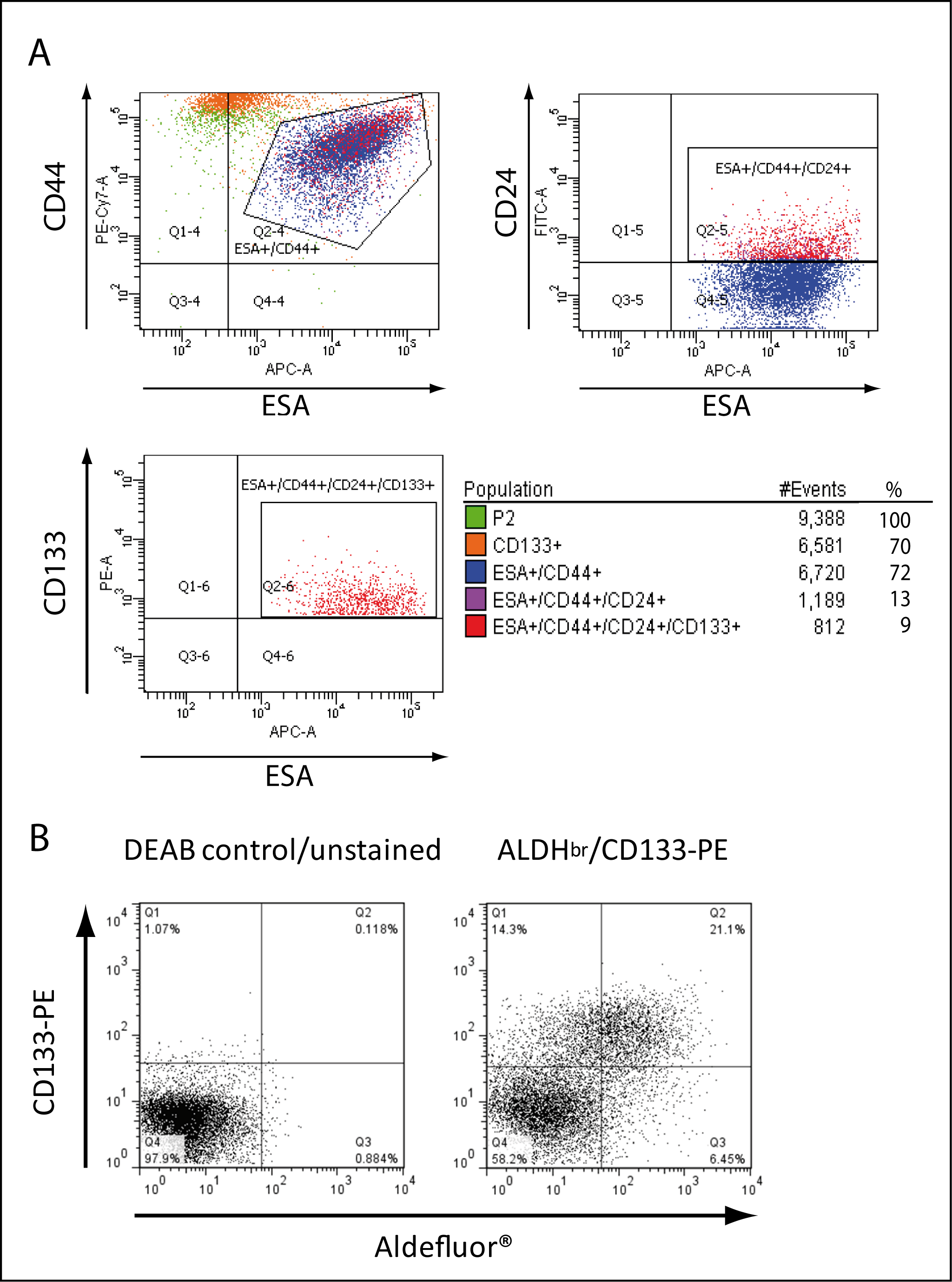

Supplement: Figure S1 — Expression and overlap of CD44/CD24/ESA/CD133 in JoPaca-1. A. Expression of putative stem cell markers CD44, CD24, ESA, and CD133 was determined by FACS using the respective antibodies conjugated to fluorescent dyes: Phycoerythrin-Cy7 to anti CD44, FITC to anti-CD24, APC to anti-ESA, and phycoerythrin to anti-CD133. Depicted here are two-dimensional dot plots showing population-overlap between ESA and CD44; ESA, CD44, and CD24 as well as ESA, CD44, CD24, and CD133. All events were normalized to the parental population (P2). B. Expression of CD133 and ALDH1-activity (ALDHbr) of JoPaca-1 cells were measured by FACS using phycoerythrin-conjugated CD133-antibody and the Aldefluor assay, respectively. Twenty one percent of JoPaca-1 cells were CD133+ and ALDH1-active. (TIF) [file pone.0048503.s001.tif]

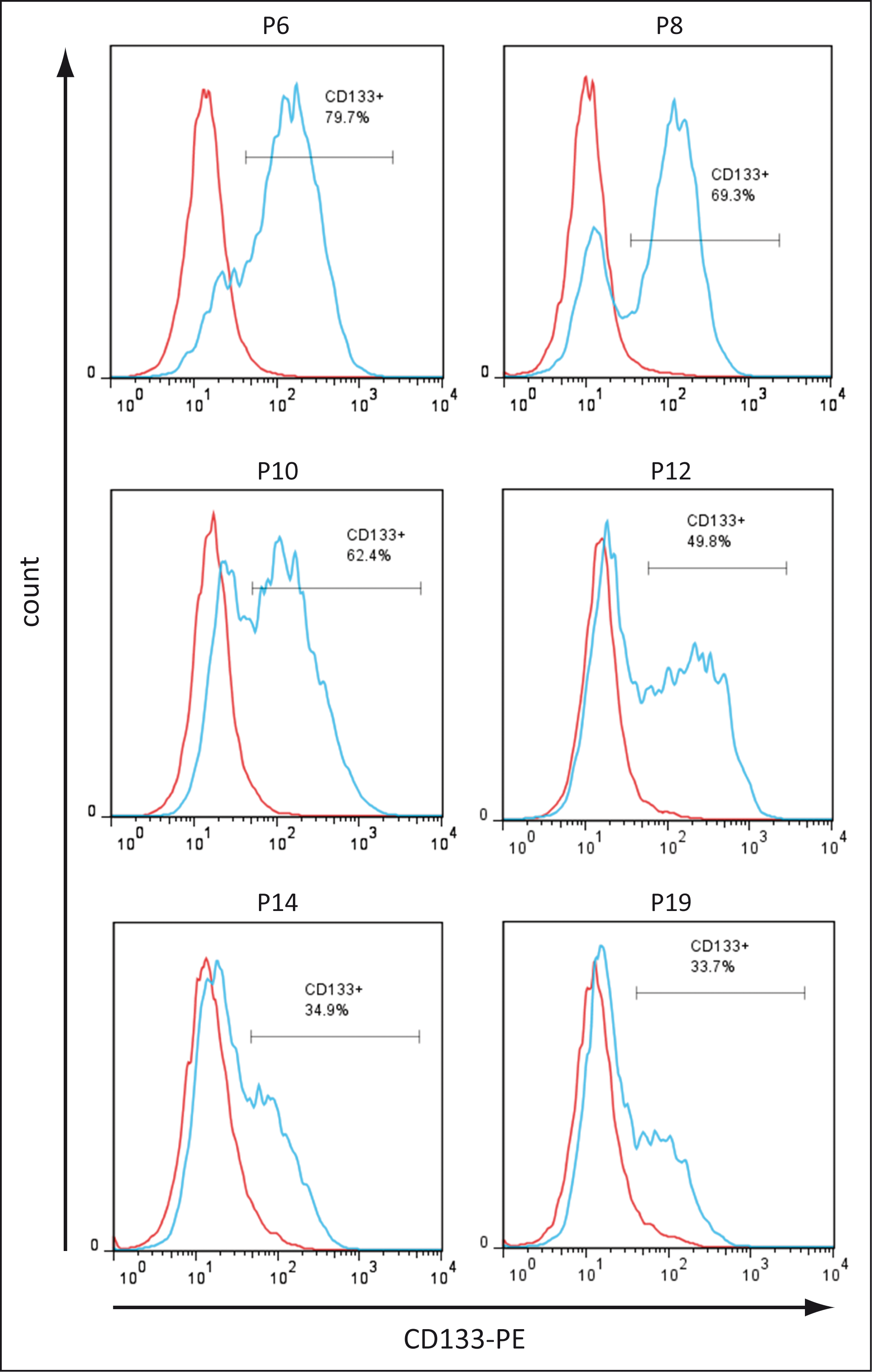

Supplement: Figure S2 — FACS histograms of CD133 content in JoPaca-1. The content of CD133+ cells in JoPaca-1 was determined in increasing passages. Histograms are shown of cells stained with phycoerythrin-conjugated CD133-antibody (blue) and unstained cells (red) determined by FACS. (TIF) [file pone.0048503.s002.tif]

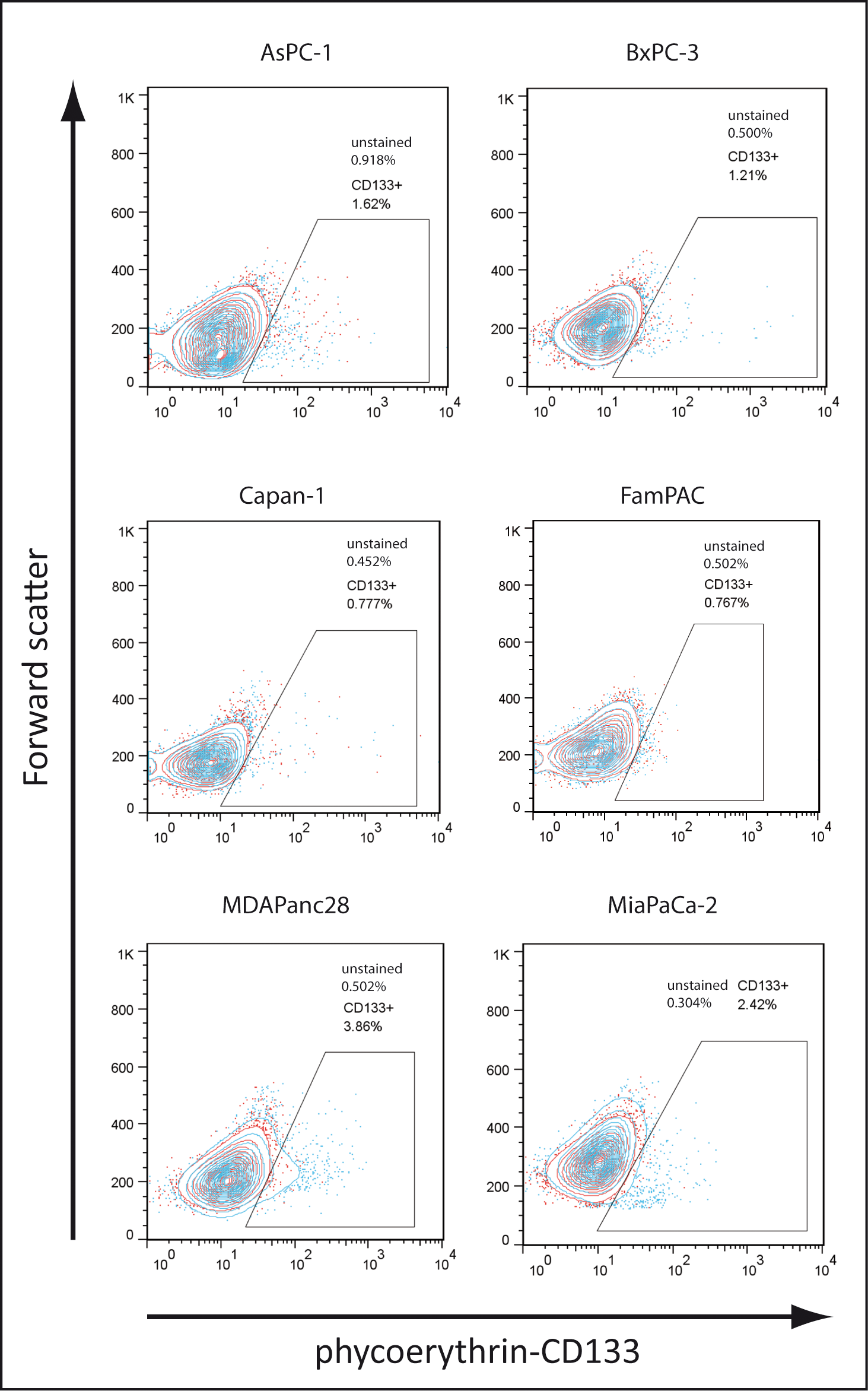

Supplement: Figure S3 — FACS contour plots of cell lines with low CD133 content. The CD133+ content of established pancreatic cell lines was measured by FACS. Depicted here are contour plots of unstained cells (red) and cells stained with phycoerythrin-conjugated CD133-antibody (blue). Outliers of the contour are shown as dots and were include in the analysis. The gate for CD133+/− discrimination was positioned close to the edge of unstained cells owing to the small fraction of CD133+ cells. To correct the resulting bias, fractions of positively gated unstained cells were considered as background and subtracted from the percentage of positively gated stained cells. Capan-1 and MDAPanc28 have close to no CD133+ cells while FamPAC and MiaPaCa-2 have a CD133+ content of about two to three percent. (TIF) [file pone.0048503.s003.tif]

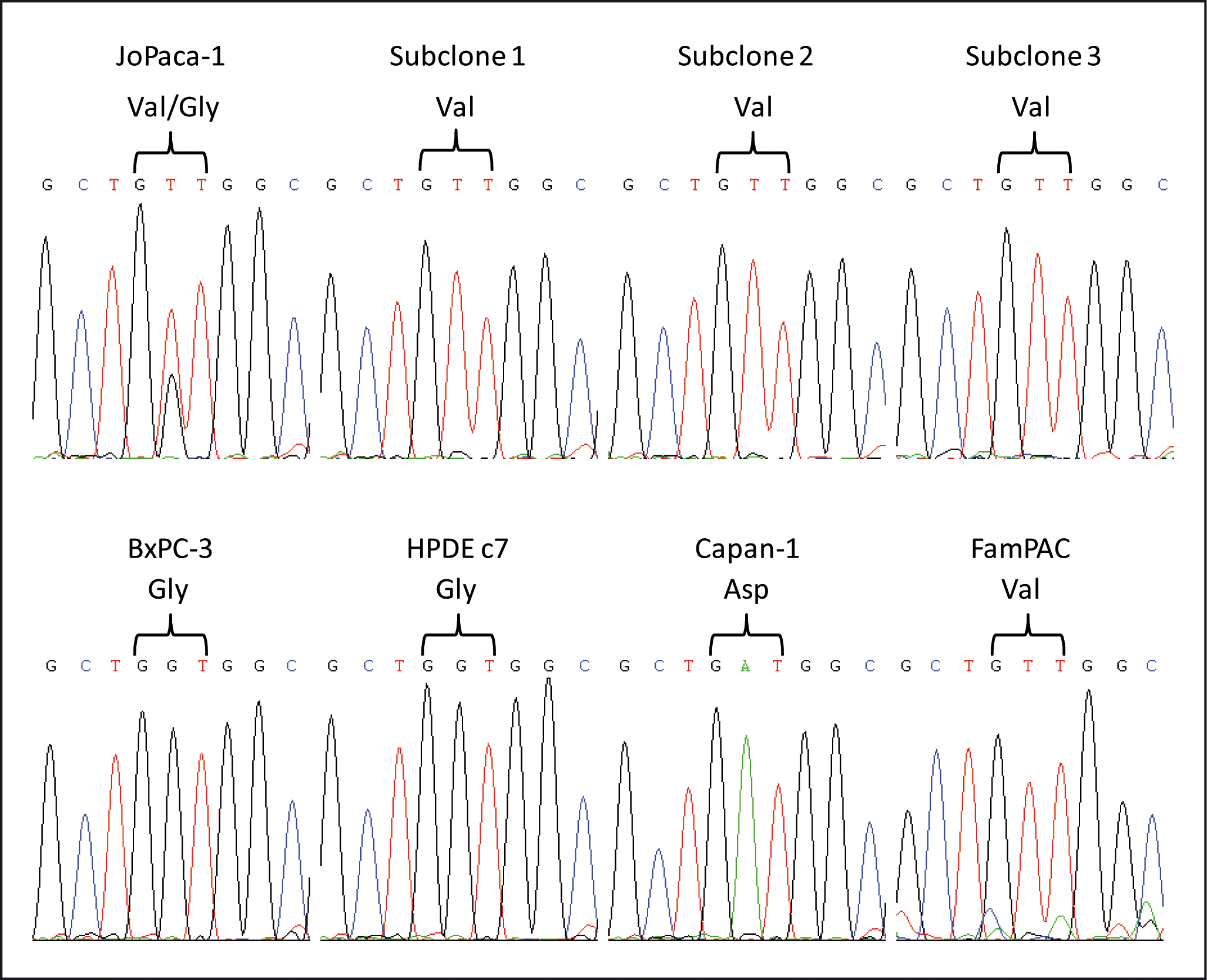

Supplement: Figure S4 — Sanger sequencing of KRAS codon 12. Electropherograms are presented for codons 11, 12, and 13 of the KRAS locus of JoPaca-1 parental cells and three JoPaca-1 subclones as well as four established cell lines. Sanger sequencing of JoPaca-1 parental cells detected both mutated (dT) and wildtype (dG) basepairs at the second position of codon 12, whereas all JoPaca-1 subclones were mutated (dA). Established cell line BxPC-3 and the pancreatic normal cells HPDE c7 are of wildtype sequence while Capan-1 and FamPAC contain mutations dA (Asp = D) and dT (Val = V). (TIF) [file pone.0048503.s004.tif]
