# Supplementary material for: Establishment and Characterization of a Highly Tumourigenic and Cancer Stem Cell Enriched Pancreatic Cancer Cell Line as a Well Defined Model System
Source: PLoS One. 2012 Nov 12;7(11):e48503. doi: 10.1371/journal.pone.0048503 (PMC3495919; doi:10.1371/journal.pone.0048503)
Supplement: Table S1 — Chromosomal aberrations observed in the karyograms of 26 JoPaca-1 cells. Listed here are all observed aberrations and their prevalence in 26 karyograms. “t” marks translocations and “i” stands for inversions. (DOCX) [file pone.0048503.s005.docx]

| **aberration** | **count** |
| --- | --- |
| t(17:5)(5;17) | 14 |
| t(7;4) | 13 |
| t(13;12)(12;13) | 8 |
| t(2;9) | 6 |
| i13 | 4 |
| t(2;20) | 3 |
| t(14;20) | 2 |
| t(17;5) | 2 |
| t(20;1)(1;20) | 1 |
| t(19;18) | 1 |
| t(13;22) | 1 |
| t(10;20) | 1 |
| t(16;20) | 1 |
| t(14;18) | 1 |
| t(12;19) | 1 |
| i14 | 1 |
| t(4;14) | 1 |
| t(14;22) | 1 |
| t(8;13)(13;8) | 1 |
| t(14;5) | 1 |
| t(1;19) | 1 |
| t(17;9) | 1 |
| t(20;2) | 1 |
| t(1;20) | 1 |
| t(11;18) | 1 |
| t(18;1) | 1 |
| t(7;8) | 1 |
| t(9;6) | 1 |
| t(18;13) | 1 |
| t(x;13) | 1 |
| t(7;13) | 1 |
| t(4;7) | 1 |
| t(14;16) | 1 |
| t(6;8) | 1 |
| t(15;4) | 1 |
| t(8;21) | 1 |
| t(13;15) | 1 |
| t(22;13) | 1 |
| t(20;12) | 1 |
| t(10;11) | 1 |
| t(2;20) | 1 |
| t(14;15) | 1 |
| t(16;17) | 1 |
| t(1;7) | 1 |
| t(6;16) | 1 |
| t(18,11)(11,18) | 1 |
