# Supplementary material for: Establishment and Characterization of a Highly Tumourigenic and Cancer Stem Cell Enriched Pancreatic Cancer Cell Line as a Well Defined Model System
Source: PLoS One. 2012 Nov 12;7(11):e48503. doi: 10.1371/journal.pone.0048503 (PMC3495919; doi:10.1371/journal.pone.0048503)
Supplement: Table S2 — Most commonly observed mutations in pancreatic cancer listed for BxPC-3 and JoPaca-1. Listed here are non-synonymous coding mutations of the top-17 mutated genes for pancreatic cancer (according to the COSMIC database) as well as BRCA1/2, PALLD and PALB2 (OMIM database) for BxPC-3 and JoPaca-1. Mutated bases are shown beneath each cell line with the respective read counts in parentheses. The degree of mutation is given in percent. SMAD4 is largely deleted in BxPC-3 and JoPaca-1. (DOCX) [file pone.0048503.s006.docx]

|  |  |  |  |  | **BxPC-3** | | | **JoPaca-1** | | |
| --- | --- | --- | --- | --- | --- | --- | --- | --- | --- | --- |
| **gene symbol** | **location** | **sequence (5'-3') cDNA** | **bases (coverage)** | **identifier** | **bases (coverage)** | **% mut.** | **aa-change** | **bases (coverage)** | **% mut.** | **aa-change** |
| KRAS | 12:25398284 | GTTGGAGCTG(G>T)TGGCGTAGGC | G/T | COSM520 | G (26) | 0 |  | G (2), T (17) | 89 | G12V |
| TP53 | 17:7579472 | GCTGCTCCCC(C>G)CGTGGCCCCT | C/G | rs1042522 | G (14) | 100 | P72R | G (19), T (1) | 100 | P72R |
| TP53 | 17:7578190 | GTGGTGCCCT(A>G)TGAGCCGCCT | A/G | COSM10758 | G (27) | 100 | Y to C | A (14) | 0 |  |
| MEN1 | 11:64572018 | GGTGATGCTG(A>G)GGGTGCTGGC | A/G | rs2959656 | G (30), C (1) | 100 | T to A | G (42) | 100 | T to A |
| ATRX | X:76937963 | TGGGAAAGAG(C>G)AGAGTTTTAC | C/G | rs3088074 | G (16) | 100 | Q to E | C (8), T(1) | 11 |  |
| APC | 5:112176756 | AATTCCAAGG(T>A)CTTCAATGAT | T/A | rs459552 | A (17) | 100 | V to D | A (9) | 100 | V to D |
| MLL3 | 7:151927021 | CATCCATACT(G>T)TGTCAGTATT | G/T | rs76778303 | G (41), T (29) | 41 | C to F | G (60), T (29) | 33 | C to F |
| MLL3 | 7:151927025 | TTACCATCCA(T>C)ACTGTGTCAG | T/C | rs77735469 | T (43), C (23) | 35 | Y to H | T (61), C (21) | 26 | Y to H |
| MLL3 | 7:151932916 | AATCGGAGCT(G>C)TTGTATTACC | G/C | rs111791757 | G (29), C (10) | 26 | V to L | G (21), C (2) | 9 | V to L |
| MLL3 | 7:151932945 | GGCCGAGGCA(G>A)GTCAAAGCTG | G/A | rs148561072 | G (30), A (15), | 33 | R to L | G (19), A (5) | 21 | R to L |
| MLL3 | 7:151935910 | ATATCCTAGG(G>A)GGCTTGGAGT | G/A | rs4024419 | G (22), A (6) | 21 | G to E | G (17), A (6) | 26 | G to E |
| SMARCA4 | 19:11123647 | CATGTCCCTG(T>G)GGTCGGTGAG | T/G | none available | T (33), G (4) | 11 | V to G | T (22), G (10) | 43 | V to G |
| BRCA1 | 17:41246481 | TGGAATAAGC(A>G)GAAACTGCCA | A/G | rs1799950 | A (20) | 0 |  | A (6), G (21) | 22 | Q to P |
| BRCA2 | 13:32906729 | CCGAAGACTA(A>C)ACGATGTAAA | A/C | CM002750 | A (28) | 0 |  | A (17) C (18) | 51 | N to H |
| BRCA2 | 13:32929387 | AATCAAGCAG(T>C)AGCTGTAACT | T/C | rs169547 | C (27) | 100 | V2466A | C (28) | 100 | V2466A |
| PALLD | 4:169606649 | CACAGTCCAA(C>A)TTCATATCTC | C/A | rs62333891 | C (23) | 0 |  | C (4), A (17) | 81 | T to N |
| PALLD | 4:169799448 | CTTCGGCCAC(A>G)GCCAGACGCC | A/G | rs62333013 | G (5) | 100 | S to G | A (2), G (14) | 88 | S to G |
| **genes with no mutations in coding region:** PALB2, CTNNB1, DAXX, ARID1A, MAP2K4, PIK3CA, STK11, PTEN, BRAF, SMAD4 (del) | | | | | | | | | | |
