# Supplementary material for: Establishment and Characterization of a Highly Tumourigenic and Cancer Stem Cell Enriched Pancreatic Cancer Cell Line as a Well Defined Model System
Source: PLoS One. 2012 Nov 12;7(11):e48503. doi: 10.1371/journal.pone.0048503 (PMC3495919; doi:10.1371/journal.pone.0048503)
Supplement: Table S3 — Selected SNPs from genome wide association studies in pancreatic cancer listed for BxPC-3 and JoPaca-1. The table lists significant non-coding variations for PDAC and their degree of mutation in the cell lines BxPC-3 and JoPaca-1. Mutations were selected from genome wide association studies up to January 2012. Sources can be identified by the first three letters of the first authors' last names: Innocenti [41], Low [42], Petersen [43], [44], Diergaarde [45], and Rizzato [46]. Mutated (mut) and wildtype (wt) bases are shown beneath each cell line with the respective read counts. The degree of mutation is given in percent. (DOCX) [file pone.0048503.s007.docx]

|  |  |  |  |  | **ancestral allel** | **BxPC-3** | | | **JoPaca-1** | | |
| --- | --- | --- | --- | --- | --- | --- | --- | --- | --- | --- | --- |
| **location (GRCh37)** | **rs number** | **source** | **associated gene(s)** | **type** |  | **mut** | **wt** | **% mut** | **mut** | **wt** | **% mut** |
| 1:190531999 | rs814951 | Inn | gene desert | intergenic | C |  | 23*C | 0 | 29*T |  | 100 |
| 11:122589092 | rs10736526 | Inn | UBASH3B | intergenic | T | 17*C | 17*T | 50 | 30*C |  | 100 |
| 12:32436409 | rs708224 | Low | BICD1 | intronic | A | 29*G |  | 100 | 23*G |  | 100 |
| 13:73896221 | rs9564966 | Pet | gene desert | intergenic | A |  | 22*A | 0 | 19*G |  | 100 |
| 14:103539669 | rs7149097 | Inn | gene desert | intergenic | A | 58*G |  | 100 | 25*G |  | 100 |
| 15:36654597 | rs8028529 | Pet, Riz | gene desert | intergenic | C | 24*T |  | 100 | 23*T |  | 100 |
| 16:13822359 | rs179619 | Inn | gene desert | intergenic | C | 24*T |  | 100 | 13*T |  | 100 |
| 16:49956194 | rs4785367 | Inn | gene desert | intergenic | T | 21*C |  | 100 | 26*C |  | 100 |
| 2:101922170 | rs6711606 | Low | RNF149 | intronic | T | 23*G |  | 100 | 16*G |  | 100 |
| 2:105378957 | rs12615966 | Low | LOC284998 | intergenic | C | 6*T | 6*C | 50 | 26*C |  | 100 |
| 2:137555224 | rs1427593 | Low | THSD7B | intergenic | T | 9*C | 13*T | 41 | 18*C |  | 100 |
| 2:235615197 | rs6736997 | Low | ARL4C | intergenic | A | 6*C | 4*A | 60 | 16*C |  | 100 |
| 5:110631265 | rs306104 | Inn | CAMK4 | intronic | T | 19*C |  | 100 | 12*C |  | 100 |
| 5:2109901 | rs6879627 | Low | LOC731559 | intergenic | T | 30*C |  | 100 | 45*C |  | 100 |
| 6:1340189 | rs9502893 | Low | FOXQ1 | intergenic | C | 58*T |  | 100 | 19*T |  | 100 |
| 6:162236075 | rs3016539 | Low | PARK2 | intronic | C | 10*T | 11*C | 48 | 17*T |  | 100 |
| 8:38469303 | rs7832232 | Low | RNF5P1 | intergenic | A |  | 22*A | 0 | 32*G |  | 100 |
| 9:136149722 | rs630014 | Pet | ABO | intronic | A | 25*G |  | 100 | 9*G |  | 100 |
| 5:1322087 | rs401681 | Pet, Low | CLPTM1L, TERT | intronic | C | 25*T | 33*C | 43 | 48*T | 1*G | 98 |
| 5:1308552 | rs4635969 | Pet | CLPTM1L, TERT, MIR4457 | close to miRNA | G |  | 37*G | 0 | 16*A | 1*T | 94 |
| 2:118879253 | rs1808458 | Inn | gene desert | intergenic | C | 16*C |  | 100 | 14*T | 1*C | 93 |
| 3:59434420 | rs1910236 | Inn | gene desert | intergenic | G | 13*A | 10*G | 57 | 12*A | 1*G | 92 |
| 5:176165086 | rs490332 | Inn | gene desert | intergenic | A | 24*G |  | 100 | 11*G | 1*A | 92 |
| 12:2091257 | rs11062040 | Inn | DCP1B | intronic | C | 11*T | 7*C | 61 | 13*T | 2*C | 87 |
| 17:70407856 | rs3744311 | Inn | gene desert | intergenic | C |  | 27*C | 0 | 13*T | 4*C | 76 |
| 16:79044649 | rs2550731 | Inn | WWOX | intronic | G | 19*C | 18*G | 51 | 13*T 1*A | 5*G | 74 |
| 8:124765702 | rs10088262 | Low | FAM91A1 | intergenic | A | 27*G |  | 100 | 13*A | 9*G | 59 |
| 7:155619733 | rs167020 | Pet | SHH | intergenic | A | 18*G | 8*A | 69 | 7*G | A*5 | 58 |
| 13:73916628 | rs9543325 | Low | gene desert | intergenic | C |  | 46*C | 0 | 20*T | 22*C | 48 |
| 13:73932114 | rs1886449 | Low | LOC730242 | intergenic | C |  | 37*C | 0 | 7*C | 9*T | 44 |
| 7:155615627 | rs172310 | Pet | SHH | intergenic | A | 26*C 1*G | 26*A | 51 | 9*C | 11*A 1*G | 43 |
| 10:103113035 | rs10883617 | Inn | BTRC | intergenic | T | 14*C |  | 100 | 6*C | 9*T | 40 |
| 22:24990213 | rs4820599 | Die | GGT1 | intronic | A |  | 36*A | 0 | 9*G | 23*A | 28 |
| 17:30877658 | rs225190 | Low | MYO1D | intronic | C |  | 15*C | 0 | 5*T | 14*C | 26 |
| 13:66481815 | rs1585440 | Low | LOC387933 | intergenic | G | 5*T | 4*G | 56 |  | 37*G | 0 |
| 17:18753870 | rs4924935 | Low | PRPSAP2 | intronic | C | 29*T | 1*C | 97 |  | 23*C | 0 |
| 7:153625843 | rs6464375 | Low | DPP6 | intronic | C | 19*T |  | 100 |  | 21*C | 0 |
| 9:136139265 | rs657152 | Pet | ABO | intronic | C | 23*A |  | 100 |  | 12*C | 0 |
| 9:136149229 | rs505922 | Pet, Low | ABO | intronic | T | 46*C |  | 100 |  | 20*T | 0 |
